# Disruption of Mouse Cenpj, a Regulator of Centriole Biogenesis, Phenocopies Seckel Syndrome

**DOI:** 10.1371/journal.pgen.1003022

**Published:** 2012-11-15

**Authors:** Rebecca E. McIntyre, Pavithra Lakshminarasimhan Chavali, Ozama Ismail, Damian M. Carragher, Gabriela Sanchez-Andrade, Josep V. Forment, Beiyuan Fu, Martin Del Castillo Velasco-Herrera, Andrew Edwards, Louise van der Weyden, Fengtang Yang, Sanger Mouse Genetics Project, Ramiro Ramirez-Solis, Jeanne Estabel, Ferdia A. Gallagher, Darren W. Logan, Mark J. Arends, Stephen H. Tsang, Vinit B. Mahajan, Cheryl L. Scudamore, Jacqueline K. White, Stephen P. Jackson, Fanni Gergely, David J. Adams

**Affiliations:** 1 Experimental Cancer Genetics, Wellcome Trust Sanger Institute, Hinxton, United Kingdom; 2 Cancer Research UK Cambridge Research Institute, Li Ka Shing Centre and Department of Oncology, University of Cambridge, Cambridge, United Kingdom; 3 Mouse Genetics Project, Wellcome Trust Sanger Institute, Hinxton, United Kingdom; 4 Genetics of Instinctive Behaviour, Wellcome Trust Sanger Institute, Hinxton, United Kingdom; 5 The Gurdon Institute and Department of Biochemistry, University of Cambridge, Cambridge, United Kingdom; 6 Molecular Cytogenetics, Wellcome Trust Sanger Institute, Hinxton, United Kingdom; 7 Wellcome Trust Center for Human Genetics, Oxford, United Kingdom; 8 Department of Radiology, Addenbrooke's Hospital, University of Cambridge, Cambridge, United Kingdom; 9 Department of Pathology, Addenbrooke's Hospital, University of Cambridge, Cambridge, United Kingdom; 10 Department of Ophthalmology and Visual Sciences, University of Iowa, Iowa City, Iowa, United States of America; 11 Bernard and Shirlee Brown Glaucoma Laboratory, Department of Ophthalmology, College of Physicians and Surgeons, Columbia University, New York, New York, United States of America; 12 Department of Pathology and Infectious Diseases, Royal Veterinary College, London, United Kingdom; Medical Research Council Human Genetics Unit, United Kingdom

## Abstract

Disruption of the centromere protein J gene, *CENPJ (CPAP, MCPH6, SCKL4)*, which is a highly conserved and ubiquitiously expressed centrosomal protein, has been associated with primary microcephaly and the microcephalic primordial dwarfism disorder Seckel syndrome. The mechanism by which disruption of *CENPJ* causes the proportionate, primordial growth failure that is characteristic of Seckel syndrome is unknown. By generating a hypomorphic allele of *Cenpj*, we have developed a mouse (*Cenpj^tm/tm^*) that recapitulates many of the clinical features of Seckel syndrome, including intrauterine dwarfism, microcephaly with memory impairment, ossification defects, and ocular and skeletal abnormalities, thus providing clear confirmation that specific mutations of *CENPJ* can cause Seckel syndrome. Immunohistochemistry revealed increased levels of DNA damage and apoptosis throughout *Cenpj^tm/tm^* embryos and adult mice showed an elevated frequency of micronucleus induction, suggesting that *Cenpj*-deficiency results in genomic instability. Notably, however, genomic instability was not the result of defective ATR-dependent DNA damage signaling, as is the case for the majority of genes associated with Seckel syndrome. Instead, *Cenpj^tm/tm^* embryonic fibroblasts exhibited irregular centriole and centrosome numbers and mono- and multipolar spindles, and many were near-tetraploid with numerical and structural chromosomal abnormalities when compared to passage-matched wild-type cells. Increased cell death due to mitotic failure during embryonic development is likely to contribute to the proportionate dwarfism that is associated with *CENPJ*-Seckel syndrome.

## Author Summary

Mutation of the gene *CENPJ* has been found to cause primary microcephaly, an inherited disorder that is characterised by severely reduced brain size. More recently, mutation of *CENPJ* has been associated with Seckel syndrome, a disorder that is characterised by a severe reduction in both brain and body size that is apparent at birth, mental retardation, and skeletal abnormalities, in addition to a number of other clinical manifestations. Here, we have generated a mouse that expresses only low levels of mouse Cenpj protein and find that it recapitulates many of the key features of Seckel syndrome. Moreover, we find that errors during the proliferation of *Cenpj^tm/tm^* cells frequently lead to abnormal numbers of chromosomes or damaged chromosomes, which is likely to be the cause of increased cell death during embryonic development and to contribute to the proportionate dwarfism that is characteristic of Seckel syndrome.

## Introduction

**Figure 1:**
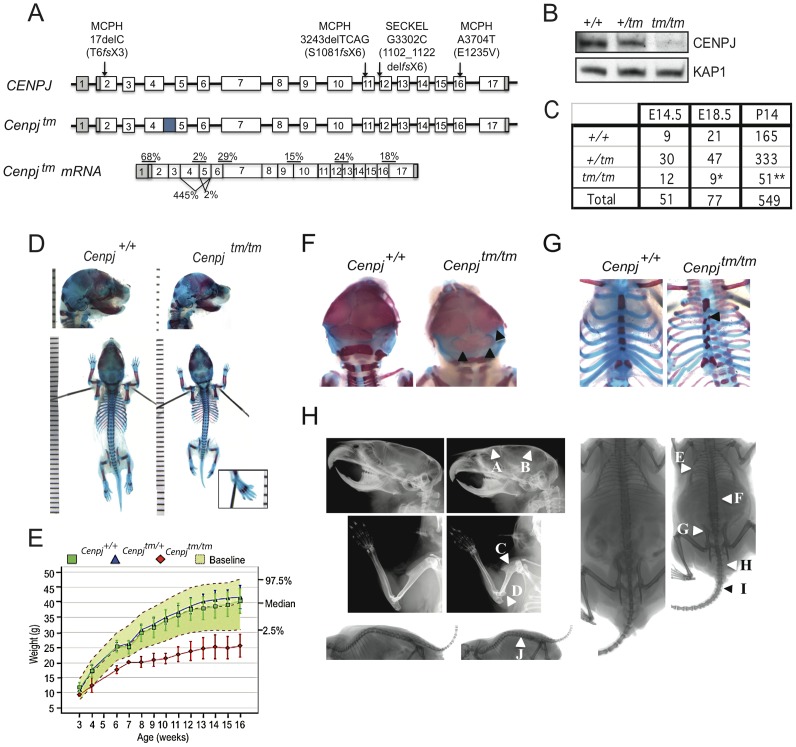
Generation of a mouse model of *CENPJ*-Seckel syndrome. A. The *CENPJ* gene spans 40 kb and comprises 17 exons. The 3′ and 5′ untranslated regions are depicted in grey. Mutations in *CENPJ* have been associated with either primary microcephaly (MCPH) or Seckel syndrome (SECKEL). The mutation in intron 11 that has been associated with Seckel syndrome results in the generation of three transcripts: one lacking exon 12, one lacking 11 and 12 and one lacking exons 11,12 and 13. Disruption of mouse *Cenpj* by insertion of a cassette (depicted by the blue square) between exons 4 and 5 results in low levels of splicing over the cassette and cryptic splicing between exons 3 and 6 or 4 and 6; the latter two transcripts are predicted to result in truncated proteins. The allele was designated *Cenpj^tm1a(EUCOMM)Wtsi^* and abbreviated to *Cenpj^tm^*. Percentages show mean expression of *Cenpj* across exon boundaries as determined by quantitative RT-PCR relative to *Gapdh* for *Cenpj^tm/tm^* relative to *Cenpj^+/+^* for RNA extracted from n = 3 murine embryonic fibroblast (MEF) lines. B. Immunoblot to show reduction in Cenpj levels in protein extracted from *Cenpj^tm/tm^* (*tm/tm*), *Cenpj^+/tm^* (+/tm), and *Cenpj^+/+^*(+/+) MEFs. KAP1 was used as a loading control. C. Table shows frequency of *Cenpj^tm/tm^* mice born from heterozygote intercrosses. *Cenpj^tm/tm^* showed partial embryonic lethality as shown by their reduced frequency at E18.5 and P14 (25% expected, *P = 0.02, **P = 0.0001, χ^2^ test). D. Representative images of E18.5 skeletal preparations of *Cenpj^+/+^* and *Cenpj^tm/tm^* embryos. Staining with alcian blue (cartilage) and alizarin red (bone). *Cenpj^tm/tm^* embryo with a sloping forehead and polysyndactylism of digit one of the left hindpaw (inset). E. Bodyweights of male *Cenpj^tm/tm^* (n = 8), *Cenpj^+/tm^* (n = 7), *Cenpj^+/+^*(n = 40) and baseline wild-type controls (n = 912) from 3–16 weeks of age. Data show that *Cenpj^tm/tm^* are significantly smaller than *Cenpj^+/+^* mice at all ages (*P* = 2.2×10^−16^, Mann-Whitney-Wilcoxon test). F. Skeletal preparations of E18.5 *Cenpj^tm/tm^* embryos showed irregular ossification of the cranium and G. sternum. H. X-Rays show that adult *Cenpj^tm/tm^* mice may present with a flatter, sloping forehead (A), mild elevation of the parietal bone (B), a short humerus with a prominent deltoid tuberosity (C), prominent medial epicondyle (D), an irregular ribcage (E), short lumbar and sacral vertebrae (F), an abnormal pelvis (G), extra sacrocaudal transitional vertebrae (H), short, abnormal/fused caudal vertebrae 2/3 – caudal vertebrae 7/8 (I) and reduced intervertebral joint space (J).

Seckel syndrome is a clinically and genetically heterogeneous primordial dwarfism disorder that is characterised by intrauterine growth retardation, postnatal dwarfism, severe microcephaly, mental retardation, a prominent curved nose and receding chin, together with other clinical abnormalities [1], [2], [3]. Mutations in five loci have been linked with Seckel syndrome: *SCKL1* and *SCKL2* are due to mutation of the genes for the DNA damage response proteins ATR and CtIP (RBBP8), respectively; *SCKL4* and *SCKL5* are due to mutation of the genes for the centrosomal proteins CENPJ (Centromere protein J, or centrosomal P4.1-associated protein, CPAP; Figure 1A) and CEP152; while the gene responsible for *SCKL3* is currently unknown [4], [5], [6], [7]. Mutations in *PCNT* (pericentrin), another centrosomal protein, have been associated with both Seckel syndrome and the overlapping dwarfism disorder, microcephalic osteodysplastic primordial dwarfism type II (MOPDII) [8], [9], [10]. Interestingly, mutations in the centrosomal proteins *CEP152* (*MCPH4*) and *CENPJ* (*MCPH6*), which are thought to interact with each other during centriole biogenesis [11], [12], have also been associated with primary autosomal recessive microcephaly, a genetically heterogeneous condition caused by mutation of one of eight loci (*MCPH1–8*; [13], [14], [15]) result in clinically indistinguishable features that include mental retardation and a severely reduced brain size of greater than two standard deviations below the average. Primary autosomal recessive microcephaly presents at birth or becomes apparent within the first few years of life [16]. Primary microcephaly is thought to be caused by a reduction in neurogenesis while the proportionate dwarfism of Seckel syndrome is thought to be the result of premature death of proliferating cells; it is not clear why different mutations in centrosomal proteins cause Seckel syndrome, MOPDII or primary microcephaly.

CENPJ is a conserved, ubiquitously expressed centrosomal protein with a key role in centriole biogenesis [17], [18], [19], [20], [21]. The centrosome is a major microtubule organizing centre in somatic cells that undergoes a duplication cycle that is tightly coupled with DNA replication (reviewed by [22] and [23]). Briefly, in G1 the centrosome is composed of a pair of loosely connected centrioles that are embedded in a proteinaceous matrix. In concert with DNA replication, a single procentriole forms next to each parental centriole during S-phase. The procentrioles continue to elongate and by the onset of mitosis two centrosomes are present, each comprising an older and younger centriole. The two centrosomes aid the formation of the poles of the bipolar spindle, the molecular machinery responsible for correct segregation of sister chromatids into daughter cells. Centrosome attachment to the poles also ensures that each daughter cell inherits a single centrosome, thus tightly regulating ploidy and centrosome numbers. Impaired centrosome duplication cycles or a failure of centrosome segregation result in abnormal centrosome numbers that in turn perturb bipolar spindle assembly and chromosome segregation [24].

*CENPJ* contains 17 exons and encodes a 1338 amino acid residue protein with a chromosomal segregation ATPase domain and a T-complex protein 10 (TCP10)-like C-terminal domain. Seckel-syndrome of a consanguineous Saudi Arabian family has been associated with a homozygous splice acceptor mutation in the last nucleotide of *CENPJ* intron 11 (Figure 1A) that results in the production of three transcripts lacking either exon 12, exons 11 and 12 or exons 11, 12 and 13 [4]. Three *CENPJ*-microcephaly mutations in three consanguineous Pakistani families have been reported to date and all are predicted to cause a truncating stop codon (Figure 1A; [14], [15]).

ATR, RBBP and CEP152 have been shown to play a role in maintaining genomic stability through regulation of the DNA damage response [5], [6], [25], however such a role has not yet been defined for CENPJ. We set out to develop a mouse model of *CENPJ*-Seckel syndrome in order to establish the mechanism by which mutation of *CENPJ* results in this subtype of primordial dwarfism. We show that the *Cenpj* hypomorphic mouse that we created recapitulates many key features of Seckel syndrome, including microcephaly with memory impairment, dwarfism from birth, and skeletal abnormalities. We further establish that wide-scale genomic instability is the likely cause of cell death within *Cenpj^tm/tm^* embryos and suggest that this contributes to the developmental phenotypes observed in *CENPJ*-Seckel patients.

## Results

### Generation and phenotyping of a *Cenpj* hypomorphic mouse

Knockout mice carrying the *Cenpj^tm1a(EUCOMM)Wtsi^* allele (Figure 1A and Figure S1A) were generated on a C57BL/6NTac; C57BL/6-Tyr^c-Brd^ background by the Sanger Mouse Genetics Project as part of the European Conditional Mouse Mutagenesis Program (EUCOMM; [26]). Correct gene targeting in founder mice was determined by a combination of standard and quantitative PCR (Figure S1). LacZ staining was detected in the brain and kidneys, while strong staining was present in the testes of mice heterozygous for the *Cenpj^tm1a(EUCOMM)Wtsi^* allele (Figure S2A).

The tm1a(EUCOMM)Wtsi gene-trap cassette that was introduced into the *Cenpj* locus is designed to truncate mRNA expression and to generate out-of-frame products following the deletion of a critical exon. Previous studies have indicated that mRNAs of certain microcephaly-associated genes are very stable [27] prompting us to perform a detailed analysis of expression and splicing at the *Cenpj^tm1a(EUCOMM)Wtsi^* locus. We generated *Cenpj^tm1a(EUCOMM)Wtsi/tm1a(EUCOMM)Wtsi^* (*Cenpj^tm/tm^*) mouse embryonic fibroblasts (MEFs; 13.5 d.p.c.) and performed SYBR Green qPCR on cDNA using primers spanning the boundaries between different exons (Figure 1A). We observed a low but detectable amount of splicing over the gene-trap cassette in *Cenpj^tm/tm^* MEFs (2.1±0.5% of wildtype exon 4–5 levels) and immunoblotting (Figure 1B) confirmed the production of low levels of apparently full-length Cenpj protein [27]. Splicing from exons 3 to 6 and 4 to 6 was detected in both *Cenpj^tm/tm^* and wildtype MEFs (Figure S2B). Between exons 3 and 6 the level of splicing detected in *Cenpj^tm/tm^* MEFs was increased relative to the levels in control MEFs (444±95%), while decreased levels of splicing were observed between exons 4 and 6 (2.1±0.5%). Using the web-based ExPASy translation tool (http://web.expasy.org/translate/) we predict that mRNAs that are spliced between exons 3–6 and exons 4–6 lead to the production of proteins truncated in exon 6 (Figure S2C). Upstream of the tm1a(EUCOMM)Wtsi cassette (exons 1–2) *Cenpj* mRNA levels were 68±19% of wildtype levels. Downstream (from exon 6 to 17) of the tm1a(EUCOMM)Wtsi cassette *Cenpj* mRNA levels were approximately 20% of the levels observed in MEFs from wildtype littermates (mean±SEM, n = 3). In summary, *Cenpj^tm/tm^* MEFs are able to produce small amounts of full-length Cenpj protein due to splicing over the tm1a(EUCOMM)Wtsi gene-trap cassette (exons 4–5) and we predict that small amounts of truncated, N-terminal Cenpj protein (corresponding to exons 1 to 3 or 1 to 4) will also be produced.

Phenotyping of mice was performed at the Wellcome Trust Sanger Institute (http://www.sanger.ac.uk/mouseportal/search?query=cenpj). *Cenpj^tm1a(EUCOMM)Wtsi/+^* intercrosses gave close to the expected Mendelian frequency (25%) of homozygote embryos at E14.5 (23.5%); however, by E18.5 this had reduced to 11.6%, suggesting that disruption of *Cenpj* causes partial embryonic lethality between E14.5 and E18.5 (χ^2^ test, *P* = 0.02; Figure 1C). The majority of runted pups identified between P0 and P21 were *Cenpj^tm/tm^* (22% of *Cenpj^tm/tm^* offspring *vs.* 4.7% for *Cenpj^+/tm^*, and 2.5% for *Cenpj^+/+^*). Stunted growth was unlikely to be the result of a major feeding problem, since milk spots were observed in the stomachs of pups of all genotypes at P0. At P14, the frequency of *Cenpj^tm/tm^* mice was not significantly different to that found at E18.5 (P14: 9.2% vs. E18.5: 11.6%; Figure 1C), suggesting that although dwarfed, *Cenpj^tm/tm^* mice are not postnatally sub-viable.

### *Cenpj*-deficiency causes intrauterine and postnatal growth retardation

One of the defining characteristics of primordial dwarfism disorders, such as Seckel syndrome, is a fetus that is small for its gestational age with postnatal growth retardation [8], [28], [29]; specifically, the Saudi Arabian *CENPJ*-Seckel kindred all have anthropometric values at least seven standard deviations below the mean [4]. *Cenpj^tm/tm^* mice showed intrauterine growth retardation (Figure 1D; mean ± SEM bodyweight at E18.5, *Cenpj^+/+^* 1.12±0.03 g, *Cenpj^tm/tm^* 0.8±0.04 g; *P* = 0.0001, t-test; crown-rump length at E18.5, *Cenpj^+/+^* 23.5±0.26 mm, *Cenpj^tm/tm^* 20.7±0.54 mm; *P* = 0.0001, t-test). From 3–16 weeks, *Cenpj^tm/tm^* mice were significantly smaller than wild-type controls (*P* = 2.2×10^−16^, Mann-Whitney-Wilcoxon test; Figure 1E). The body weight of adult *Cenpj^tm/tm^* animals was 64% of wild-type controls (mean ± SEM bodyweight at 16 weeks, *Cenpj^+/+^* 39.1±1.35 g, *Cenpj^tm/tm^* 25.4±1.34 g; *P* = 9.3×10^−11^, t-test; Figure 1E) and length was 76% of controls (mean ± SEM nose-to-tail base length at 14 weeks, *Cenpj^+/+^*10.4±0.05 cm, *Cenpj^tm/tm^* 7.9±0.07; *P* = 2.2×10^−16^, t-test; Figure S2D).

### Skeletal abnormalities and abnormal ossification of bone from *Cenpj^tm/tm^* mice

The skeletal abnormalities of Seckel syndrome associated with an intron 11 mutation in *CENPJ* include a receding chin, high forehead and prominent nasal spine [4]. Although the facial features of the *CENPJ*-Seckel kindred (two siblings and three cousins) were strikingly similar, the skeletal survey of sibling one was largely normal and that of sibling two revealed 11 ribs instead of 12 and a steep acetabular roof [4]. These findings highlight the fact that the same mutation results in clinical heterogeneity and prompted us to perform a thorough skeletal analysis of *Cenpj^tm/tm^* embryos and adult mice; *Cenpj^tm/tm^* embryos had significantly smaller skulls (Figure 1D and 1H; mean ± SEM at E18.5: skull length, *Cenpj^+/+^* 9.8±0.12 mm, *Cenpj^tm/tm^* 9.3±0.17 mm, *P* = 0.0379; inner canthal distance *Cenpj^+/+^* 3.22±0.05 mm, *Cenpj^tm/tm^* 2.96±0.09 mm, *P* = 0.0198) and adult mice presented with a flatter, sloping forehead and mild elevation of the parietal bone compared to controls (Figure 1H; 16 weeks of age, n = 8 males and n = 7 females). Although the Saudi Arabian *CENPJ*-Seckel kindred do not have clinodactyly (curvature of the fifth finger), it is a frequently reported characteristic of Seckel patients [1], [4], [29], [30]. We did not observe clinodactyly in *Cenpj^tm/tm^* mice, however we noted polysyndactylism of the first digit of the left hind paw in 2/9 *Cenpj^tm/tm^* embryos (Figure 1D, inset), which has also been reported in mutant *Pcnt* (pericentrin) mice [31]. Furthermore, retarded ossification and decreased bone age is reported in the majority of cases of Seckel syndrome, although this clinical abnormality was not specifically addressed for the *CENPJ*-Seckel kindred [1]. A higher proportion of *Cenpj^tm/tm^* embryos showed incomplete or irregular ossification of the parietal and occipital bones when compared to controls (Figure 1F; 3/5 *Cenpj^tm/tm^*, 1/38 *Cenpj^+/tm^*, 1/13 *Cenpj^+/+^*). In addition, a subset of Seckel patients, including a *CENPJ*-Seckel patient, have 11 ribs instead of the usual 12 [4], [30], [32]. Although all ribs were present in *Cenpj^tm/tm^* embryos, we noted that the attachment of the ribs to the sternum followed an irregular pattern that closely corresponded to the asymmetrical distribution of ossification centers along the sternum (Figure 1G; 3/5 *Cenpj^tm/tm^*). Adult *Cenpj^tm/tm^* mice displayed an irregular ribcage, with crowding of the ribs (Figure 1H; 9/15). Moreover, a subset of Seckel patients have been reported to have bilateral dislocation of the hips and elbows, with a decreased range of motion at the elbows [1]. While we did not find any evidence of dislocation we noted that the humeri of adult *Cenpj^tm/tm^* mice were anatomically disproportionate when compared with those of wild-type mice; the deltoid tuberosities were closer to the greater tubercle when normalized to humeri length (mean±SEM.: right humeri, *Cenpj^+/+^* 49.6±0.28%, *Cenpj^tm/tm^* 47.1±0.93%; *P* = 0.02, t-test; Figure 1H), and humeri were sometimes bowed (6/15) with a very prominent medial epicondyle (11/15; Figure 1H). Furthermore, all adult *Cenpj^tm/tm^* mice (15/15) displayed an abnormal pelvis that was wider at the iliac crests (Figure 1H, iliac crest normalised to ischiac, mean±S.E.M.: *Cenpj^+/+^* 69.7±1.18%, *Cenpj^tm/tm^* 60.8±1.72%; *P* = 0.0003) and sometimes asymmetrical. Finally, we observed that all *Cenpj^tm/tm^* mice had a reduced intervertebral joint space in the lumbar and caudal regions (Figure 1H). In general, lumbar and sacral vertebrae were shorter and *Cenpj^tm/tm^* mice had one to two extra sacrocaudal transitional vertebrae as a result. In 13/15 *Cenpj^tm/tm^* mice, Caudal 2/3 – Caudal 7/8 were abnormal in morphology and fused (Figure 1H).

### Neuropathological abnormalities and memory impairment

**Figure 2:**
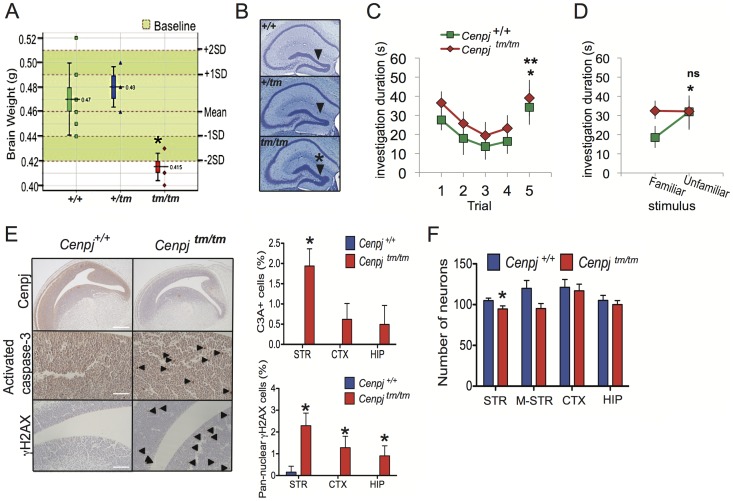
Neuropathological abnormalities. A. *Cenpj^tm/tm^* mouse brain weights were two standard deviations below that of control mice (n = 144 baseline control mice). *P = 0.0002, t-test, *Cenpj^tm/tm^*, n = 6 and *Cenpj* ^+/+^ n = 10. The lower whisker extends to the lowest datum still within 1.5 Inter-quartile range (IQR) of the lower quartile. The upper whisker extends to the highest datum still within 1.5 IQR of the upper quartile B. The dentate gyrus was significantly shorter in *Cenpj^tm/tm^* mice (n = 3) when compared to *Cenpj* ^+/+^ control mice (n = 30), *P = 0.01, t-test. Scale bar 1 mm C. Social recognition test. When tested for habituation-dishabituation, both *Cenpj^tm/tm^* (n = 7) and *Cenpj* ^+/+^ (n = 7) mice recognized a novel stimulus mouse as shown by a decline in investigation time over the first four trials that was recovered on trial five, when they were exposed to a novel mouse (trial four *vs.* trial five, * *P* = 0.0033 and ** *P* = 0.0014, two-way ANOVA followed by post-hoc t-test). D. A discrimination test was performed 24 h later the habituation-dishabituation test. When given a choice between the familiar (same stimulus animal used for trials one to four) and a new unfamiliar mouse 24 h later, *Cenpj^tm/tm^* mice could not discriminate as shown by the similar investigation time for both stimulus animals (*Cenpj* ^+/+^ *P* = 0.0326, C*enpj^tm/tm^ P* = 0.957, t-test). E. Representative images of immunohistochemical stainings of E14.5 embryo sections. Cenpj was highly expressed in areas of active neurogenesis within the telencephalon. Scale bar 400 µm. There was a generalized increase in cleaved (activated) caspase-3-positive (scale bar 100 µm) and Ser139-phosphorylated H2AX (γH2AX; scale bar 200 µm) cells throughout embryos, images of striatum are shown.. The number of cells positive (as a percentage of total in two different 75 µm^2^ areas) for cleaved (activated) caspase-3 (C3A+) and pan-nuclear Ser139-phosphorylated H2AX (γH2AX) was increased in areas of active neurogenesis within the striatum and cortex. *P<0.05; Mann-Whitney with continuity correction, *Cenpj^tm/tm^* n = 3 and *Cenpj* ^+/+^ n = 3. Data shows mean and SEM. F. Neuron densities were counted in three different areas (75 µm^2^) of active neurogenesis for each of the striatum (STR), cortex (CTX) and pro-hippocampus (HIP) and three areas of 150 µm^2^ in the mid-striatum (M-STR) of E14.5 embryos, *P = 0.0008, t-test, *Cenpj^tm/tm^* n = 3 and *Cenpj* ^+/+^ n = 3. Data shows mean and SEM.

Microcephaly is one of the defining characteristics of Seckel syndrome [1]. Microcephaly has been clinically defined as a head circumference of at least two standard deviations below the normal range; and in the case of Seckel syndrome associated with mutations in intron 11 of *CENPJ*, head circumference is seven standard deviations below the mean [4], [33]. The average *Cenpj^tm/tm^* mouse brain weight was two standard deviations below that of control mice (Figure 2A; *P* = 0.0002, t-test). Although the two and four year-old siblings with *CENPJ*-Seckel syndrome described to date had relatively normal magnetic resonance imaging (MRI), cranial MRI of adult patients with Seckel syndrome has revealed several neuroanatomical abnormalities aside from a reduction in brain volume [4], [32], [34], [35]. We therefore assessed the area of brain regions and the thickness of the neuronal layers of the adult mouse brain (16 weeks; Figure S3). Although the patterning of the hippocampal layers appeared normal, the length of the dentate gyrus was significantly reduced in *Cenpj^tm/tm^* when compared to *Cenpj* ^+/+^ control mice (mean±SEM.: *Cenpj^+/+^* 4380±64 µm, *Cenpj^tm/tm^* 3797±181 µm, *P* = 0.01, t-test; Figure 2B). The average thickness of the cortex, which is often reduced with mutation of microcephaly genes in mice [36], [37], and of the molecular, striatum radiatum and oriens layers of the hippocampus were not significantly different to wild-type controls. Similarly, the total areas of the hippocampus, corpus callosum and dorsal third ventricle were unchanged as was the total internal length of the pyramidal cell layer.

History was suggestive of normal cognitive and motor development for four of the five cases within the *CENPJ*-Seckel kindred while one patient clearly had intellectual impairment (IQ 60; MRI not performed [4]). Since the hippocampus is involved in learning and memory formation and since Seckel patients generally display learning impairments [3], [34], we performed a social recognition test with *Cenpj^tm/tm^* and control animals [38], [39], [40]; Figure 2C and 2D). Thus, on day one, mice were tested for habituation-dishabituation: male mice were presented with a novel, anaesthetized stimulus mouse and the time of investigation was recorded. Mice were then given a 10 minute resting period before this was repeated a further three times with the same stimulus mouse. On the fifth trial, mice were presented with an unfamiliar stimulus mouse (Figure 2C). Both *Cenpj^tm/tm^* (n = 7) and *Cenpj* ^+/+^ (n = 7) mice recognized and habituated to the novel stimulus mouse, as there was a decline in investigation time over the first four trials that was recovered on trial five (Figure 2C, two-way ANOVA, repeated measures for *trial* F_4,48_, = *P*<0.001, effect for *genotype* F_1,48_ = 0.5482, *P* = 0.433, *interaction* F_4,48_ = 0.09258, *P* = 0.9844), when they were exposed to a novel mouse (Figure 2C. Trial four *vs.* trial five, *P* = 0.0033 and *P* = 0.0074, post-hoc t-test). These data suggest that olfaction in *Cenpj^tm/tm^* mice is not markedly affected. Twenty-four hours after the habituation-dishabituation test, a discrimination-based olfactory memory test was performed. When given a choice between the familiar (same stimulus animal used for trials one to four) and a new unfamiliar mouse, *Cenpj* ^+/+^ animals spent less time investigating the familiar mouse than the unfamiliar one (Figure 2D. *P* = 0.0326, t-test). However, *Cenpj^tm/tm^* mice were less able to recognize the familiar from the unfamiliar animal as shown by the similar investigation time for both stimulus animals (Figure 2D. *P* = 0.957, t-test; Normalized discrimination *Cenpj* ^+/+^ vs *Cenpj^tm/tm^*, *P* = 0.0417). In summary, short-term memory and olfaction appear to be unaffected in *Cenpj^tm/tm^* mice, however long-term memory was significantly impaired. All other tests of neurological function were normal, including open field, grip strength, modified SHIRPA (the SmithKline Beecham, Harwell, Imperial College, Royal London Hospital, phenotype assessment is a set of behavioural tests designed to test muscle, cerebellar, sensory and neuropsychiatric function), auditory brainstem response and hot plate assessment.

### Increased DNA damage, apoptosis, and reduced neuron density in *Cenpj* *^tm/tm^* mice

All of the genes associated with Seckel syndrome have so far been shown to result in defective DNA damage responses and a lowered apoptotic threshold [5], [6], [7], [25], [41]. To test whether *Cenpj*-deficiency is associated with elevated levels of DNA strand breaks and/or apoptosis, we performed immunohistochemical staining of E14.5 embryos. Phosphorylation of histone H2AX on serine 139 (γH2AX) by the ATR, DNA-PK or ATM kinases occurs at sites flanking DNA strand breaks and enhances the recruitment of DNA repair proteins to sites of damage [42]; if the damage is irreparable then the cell death cascade is normally activated [43]. Compared to controls, there was a general increase in the number of γH2AX-positive cells throughout *Cenpj^tm/tm^* embryos (Figure S4) and this was most pronounced in the developing telencephalon (Figure 2E; mean±SEM. γH2AX-positive cells as a percentage of total: striatum *Cenpj* ^+/+^ 0.47±0.2%, *Cenpj^tm/tm^* 2.3±0.3%, *P* = 0.004; cortex *Cenpj* ^+/+^ 0.0%, *Cenpj^tm/tm^* 1.3±0.3%, *P* = 0.01; pro-hippocampus *Cenpj* ^+/+^ 0.0%, *Cenpj^tm/tm^* 0.9±0.3%, *P* = 0.03. Mann-Whitney with continuity correction). Similarly, there was an increase in the number of cleaved caspase-3-positive cells throughout *Cenpj^tm/tm^* embryos (Figure S4), although this was most pronounced in areas of active neurogenesis (as determined by Ki67 staining; Figure S4) within the telencephalon, where Cenpj was most highly expressed (Figure 2E; mean±S.E.M. cleaved caspase-3-positive cells as a percentage of total cells: striatum *Cenpj* ^+/+^ 0.0%, *Cenpj^tm/tm^* 1.9±0.2%, *P* = 0.001; cortex *Cenpj* ^+/+^ 0.0%, *Cenpj^tm/tm^* 0.6±0.2%, *P* = 0.05; pro-hippocampus *Cenpj* ^+/+^ 0.0%, *Cenpj^tm/tm^* 0.5±0.3%, *P* = 0.14, t-test). There was no detectable difference in the patterns of cellular proliferation between *Cenpj^tm/tm^* and *Cenpj* ^+/+^ embryos when examined using the marker Ki67 (Figure S4).

Consistent with increased levels of apoptosis in the developing telencephalon of *Cenpj^tm/tm^* embryos, reports of fetal stage Seckel syndrome (loci responsible unknown) have shown reduced neuron density and disorganization of cortical layers at 30 weeks gestation [28], [29]. We therefore quantified neuron densities in areas of active neurogenesis within the telencephalon (areas of Ki67-positive staining; Figure S4) and in the mid-striatum of embryos during mid-neurogenesis (E14.5) and found that, in general, the number of neurons were decreased in *Cenpj^tm/tm^* embryos and the reduction was significant for the striatum (Figure 2F; mean±SEM. for n = 3 (average of two different 75 µm^2^ areas) in striatum: *Cenpj* ^+/+^ 104.9±3.1, *Cenpj^tm/tm^* 94.7±3.8, *P* = 0.0008, t-test).

### Delayed puberty of female *Cenpj^tm/tm^* mice

**Figure 3:**
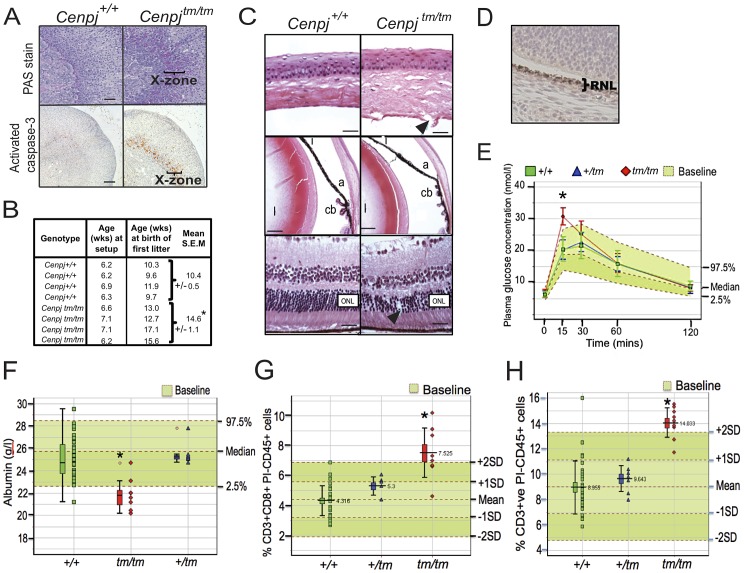
Delayed onset to puberty and ocular, endocrine, haematological, and plasma abnormalities. A. Periodic acid-Schiff (PAS) staining and cleaved (activated) caspase-3 immunostaining of adrenal sections from 16 week-old virgin female *Cenpj^tm/tm^* mice (n = 3) confirmed corticomedullary pigmentation and ongoing apoptosis in the X-zone, respectively (representative images, scale bars 100 µm). B. Breeding records of *Cenpj^tm/tm^* females set up with *Cenpj^tm/tm^* males at 6–7 weeks of age showed that *Cenpj^tm/tm^* females produce their first litter around four weeks later than *Cenpj* ^+/+^ females. *P = 0.012, t-test. C. Top panel shows normal cornea from a *Cenpj* ^+/+^ mouse. *Cenpj^tm/tm^* mice had disruption of the Descemet's membrane and corneal endothelium (arrow). Middle panel shows normal anterior segment from a *Cenpj* ^+/+^ mouse. The angle was displaced anteriorly in eyes from *Cenpj^tm/tm^* mice and ciliary process morphology was abnormal. (a, angle; i, iris; cb, ciliary body; l, lens). Bottom panel shows normal retina from a *Cenpj* ^+/+^ mouse eye. The retina photoreceptor cells of *Cenpj^tm/tm^* mice were reduced in number and showed columnar disorganized (arrow). (ONL, outer nuclear layer). D. Immunohistochemical staining for Cenpj in *Cenpj* ^+/+^ embryo eye (E14.5; RNL retinal neuroblast layer). E. Intra-peritoneal glucose tolerance test to show that female *Cenpj^tm/tm^* mice have a 15 minute delay in response to glucose challenge (n = 4 *Cenpj^tm/tm^* vs. n = 32 *Cenpj* ^+/+^, *P = 2×10^−5^, t-test). Graph also shows n = 9 *Cenpj^tm/+^* and n = 670 baseline wildtype controls. F. Plasma albumin levels were decreased in *Cenpj^tm/tm^* males (n = 8 *Cenpj^tm/tm^* vs. n = 35 *Cenpj* ^+/+^, *P = 4.9×10^−5^, t-test). Graph also shows n = 7 *Cenpj^tm/+^* and n = 768 baseline wildtype controls. G. Flow cytometric analysis of peripheral blood leukocytes in *Cenpj^tm/tm^* mice revealed an increase in the number of CD8+CD3+ and H. total CD3+ cells. Data shows total counts per 30 000 propidium-iodide (PI) negative, CD45-positive cells from male mice. For n = 9 *Cenpj^tm/tm^* vs. n = 30 *Cenpj* ^+/+^: CD3+CD8+ *P = 0.0002 and CD3 *P = 2.9×10^−5^, Mann-Whitney-Wilcoxon test. Graphs also show n = 7 *Cenpj* ^+/tm^ and n = 356 baseline wildtype controls. For all ‘Box and Whisker’ plots, the lower whisker extends to the lowest datum still within 1.5 Inter-quartile range (IQR) of the lower quartile. The upper whisker extends to the highest datum still within 1.5 IQR of the upper quartile.

Several clinical reports of patients with Seckel syndrome have described precocious puberty or premature thelarche [44], [45]. It is not yet known whether the sexual development of patients with Seckel syndrome associated with *CENPJ* mutations is normal since the cases described so far report the phenotype of infants [4]. A thorough histopathological analysis of adult male and female *Cenpj^tm/tm^* and *Cenpj* ^+/tm^ mice (n = 3 of each gender and genotype at 16 wks) revealed several anomalies, including corticomedullary pigmentation in the adrenals of female *Cenpj^tm/tm^* mice (Figure 3A). Corticomedullary pigmentation is associated with ‘X-zone’ degeneration in female mice, a sex hormone-dependent change that occurs during puberty in virgin females and is often complete by 16 weeks in C57BL/6 mice, or earlier in pregnant females [46]. By using cleaved-caspase 3 as a marker of apoptosis, we found that the adrenals of virgin *Cenpj^tm/tm^* female mice have pronounced and ongoing X-zone degeneration at 16 weeks when compared to virgin *Cenpj* ^+/+^ females (Figure 3A). Wild-type C57BL/6 female mice reach sexual maturity at around 6–7 weeks of age. These findings suggest that, in contrast to Seckel syndrome patients, puberty is delayed in *Cenpj^tm/tm^* female mice. In support of this, breeding records of females set up with *Cenpj^tm/tm^* males at 6–7 weeks of age showed that *Cenpj^tm/tm^* females produce their first litter around four weeks later than *Cenpj* ^+/+^ females (*P* = 0.012, t-test; Figure 3B). There were no morphological differences in the reproductive tract of male or female *Cenpj^tm/tm^* animals when examined at 16 weeks of age (data not shown).

### Abnormal development and structural abnormalities of the eye in *Cenpj^tm/tm^* mice

Although not reported for the *CENPJ*-Seckel kindred [4], several individuals that have been clinically diagnosed with Seckel syndrome have ocular defects, such as spontaneous lens dislocation, myopia, astigmatism, and retinal degeneration [47] [48]. A higher proportion of *Cenpj^tm/tm^* embryos had secondary anophthalmia (E18.5, 0/13 *Cenpj^+/+^*, 1/38 *Cenpj* ^+/tm^ and 1/5 *Cenpj^tm/tm^*) and a higher proportion of *Cenpj^tm/tm^* pups still had their eyes closed at P14 (5/28 *Cenpj^tm/tm^* vs. 1/46 wild-type). At 16 weeks of age, histological analysis of the eyes from *Cenpj^tm/tm^* mice showed various structural abnormalities. In the anterior segment, the corneal endothelium and Descemet's membrane was occasionally broken (Figure 3C). The anterior chamber was of normal depth but the angle was anteriorly displaced in some cases (Figure 3C). Significant cataracts were not observed in *Cenpj^tm/tm^* mice but the iris showed adhesions to the lens and its base was anteriorly shifted in relation to the ciliary body (Figure 3C). Also, the ciliary body processes were spaced far apart or blunted in *Cenpj^tm/tm^* animals, and in some cases, ciliary process morphology was abnormal (Figure 3C). In the retina, the photoreceptor nuclei were variably reduced in number and columns were loosely packed or disorganized in *Cenpj^tm/tm^* animals (Figure 3C). Other cell layers, including the retinal ganglion, inner nuclear, and retinal pigment epithelium appeared normal, and the optic nerve did not show thinning. At E14.5, Cenpj was highly expressed in the retina neuroblast layer, where cells are rapidly differentiating and proliferating, but not in the inner retinal ganglion cell progenitor layer (Figure 3D).

### Delayed response to glucose challenge in *Cenpj^tm/tm^* mice

During routine phenotyping, mice were subject to an intra-peritoneal glucose tolerance test, in which mice were fasted for 16 hours, a bolus of glucose was administered intraperitoneally and blood glucose concentration was monitored for 2 hours. Fifteen minutes after administration of glucose, 4/4 female *Cenpj^tm/tm^* mice (*Cenpj* ^+/+^ 20.5±0.7 mmol/l, *Cenpj^tm/tm^* 30.7±1.60 mmol/l, *P* = 2×10^−5^, t-test) and 2/5 male *Cenpj^tm/tm^* had blood glucose levels greater than or equal to the 97.5^th^ centile of baseline controls (n = 670 females, n = 669 males), although this had returned to normal by 30 minutes (Figure 3E).

### *Cenpj*-deficiency is associated with karyomegaly of cardiomyocytes in young mice

Despite being one of the less frequently reported characteristics of Seckel syndrome, there are numerous case-reports of severe cardiac anomalies in Seckel syndrome patients [49], [50], [51], [52], [53]. Strikingly, the majority of 16-week old *Cenpj^tm/tm^* mice (5/6) and only 1/6 *Cenpj* ^+/tm^ and 0/4 wildtype mice showed disorganization of cardiomyocytes with an increased incidence of karyomegaly and multinucleate cells, predominantly within the interventricular septum, papillary muscle and inner myocardium (Figure S2E). Cardiomyocyte karyomegaly has previously been observed in wild-type mice [54] where it may be associated with reparative processes [55] and may represent polyploidy [56]. Although the incidence and extent of karyomegaly was noticeably increased in hearts from *Cenpj^tm/tm^* mice compared to wildtype animals in this study, there was no evidence of fibrosis (consistent with previous cardiac damage) based on trichrome staining or alterations in apoptosis or proliferation (cleaved caspase-3 and Ki67, respectively; data not shown). Interestingly, the preponderance of karyomegaly in cardiomyocytes, hepatocytes and cells of the Harderian glands was increased in aged *Cenpj^tm/tm^* mice (13-month old) when compared to age-matched control (Figure S2E).

### Hypoalbuminemia of *Cenpj^tm/tm^* mice

Clinical chemistry was performed on animals at 16 weeks of age. Albumin levels were generally decreased in *Cenpj^tm/tm^* mice of both genders compared to controls, and this was statistically significant for males (mean±S.E.M, *Cenpj* ^+/+^ 25.1±0.31, *Cenpj^tm/tm^* 21.9±0.52, *P* = 4.9×10^−5^, t-test; Figure 3F).

### Increased levels of CD3^+^CD8^+^ T cells in *Cenpj^tm/tm^* mice

Flow cytometric analysis of peripheral blood leukocytes at 16 weeks of age revealed a marked increase in the frequency of the CD8 T cell subset (CD3^+^ CD8^+^) in both genders of *Cenpj^tm/tm^* mice compared to wild-type controls (mean±S.E.M Males: CD8^+^CD3^+^ gated on PI^−^ CD45^+^: *Cenpj* ^+/+^ 4.3%±0.18, *Cenpj^tm/tm^* 7.5%±0.55, *P* = 0.0002 Mann-Whitney-Wilcoxon test; Figure 3G). Furthermore, the increase in the proportion of the peripheral blood CD8 T cell population was reflected in a concomitant increase in the frequency of total T cells in *Cenpj^tm/tm^* mice (mean±S.E.M, CD3^+^ gated on PI^−^ CD45^+^: *Cenpj* ^+/+^ 9%±0.39, *Cenpj^tm/tm^* 14%±0.41, *P* = 2.9×10^−5^, Mann-Whitney-Wilcoxon test; Figure 3H). The frequency of CD4 T cells in the peripheral blood was unaffected (data not shown).

### Increased genomic instability in embryonic fibroblasts from *Cenpj*-deficient mice

**Figure 4:**
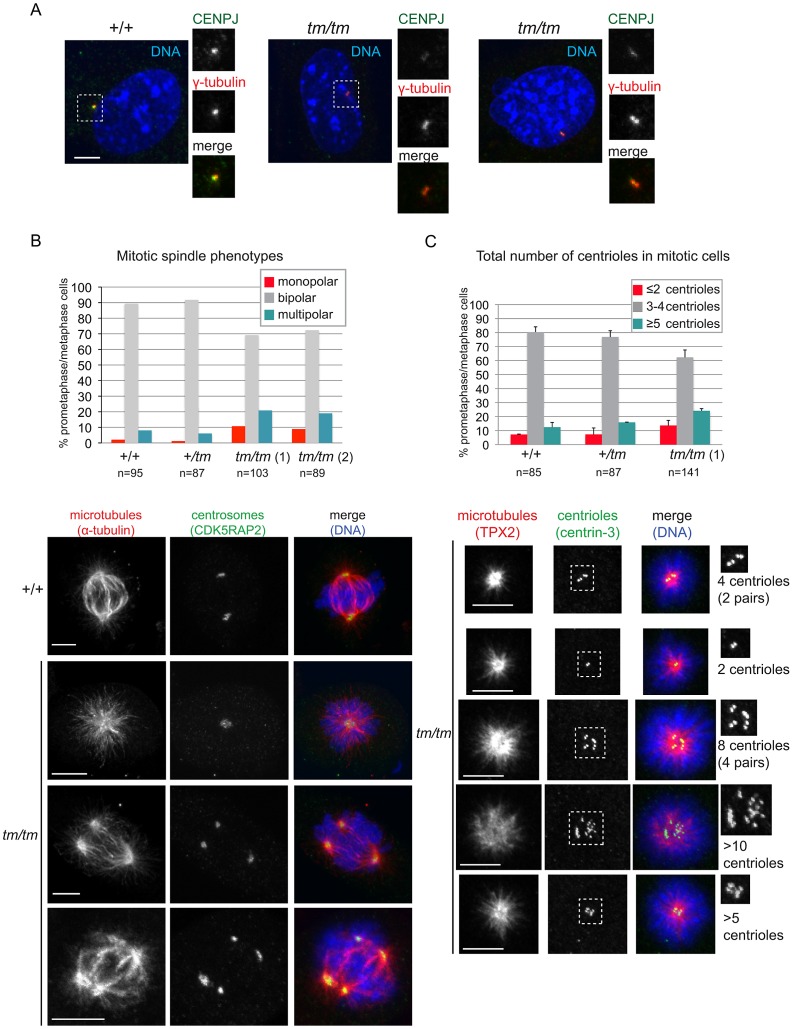
Centrosome and mitotic spindle abnormalities are elevated in *Cenpj*-deficient cells. A. Images show examples of Cenpj staining in centrosomes of *Cenpj* ^+/+^ and *Cenpj^tm/tm^* mouse embryonic fibroblasts (MEFs). Cells were stained with antibodies against Cenpj (green in merge) and the centrosomal protein γ-tubulin (red in merge). Framed areas are shown at higher magnification. B. Graph shows mitotic spindle phenotypes in MEFs derived from *Cenpj* ^+/+^, *Cenpj* ^+/tm^ and two independent *Cenpj^tm/tm^* embryos (littermates, +/+ MEFs passage 4, +/tm and *tm/tm* MEFs passage 3): *tm/tm* (1) and *tm/tm* (2). Number of mitotic cells scored are shown for each genotype. Examples for monopolar and multipolar spindle are shown. Note cell on bottom panels forming a bipolar spindle by clustering supernumerary centrosomes. Cells were stained with antibodies against α-tubulin (green in merge) and the centrosomal protein, Cdk5RAP2 (red in merge). C. Graph shows centriole numbers in mitotic MEFs of indicated genotypes (littermates, +/+ MEFs passage 4, +/tm and *tm/tm* MEFs passage 3). Cells were arrested in mitosis with monastrol that caused monopolar spindle formation and facilitated visualization of centrioles. Note that mitotic cells should normally contain a total of 4 centrioles, but even in wild-type cells we occasionally detect 3 centrioles probably due to insufficient spatial resolution, so 3 or 4 centrioles were considered a single class. Data were collected from two independent experiments; bars show mean ±SD, number of mitotic cells scored are shown for each genotype. Images below depict examples for cells with different centriole numbers (top cell with 4 centrioles is normal, all other cells have too few or too many centrioles). Cells were stained with antibodies against the microtubule-binding protein Tpx2 (green in merge) and the centriolar protein, centrin-3 (red in merge). Framed areas are shown at higher magnification. Scale bars = 5 µm.

CENPJ depletion in cultured cells has been reported to impair centriole assembly, disrupt centrosome integrity and lead to the formation of monopolar and multipolar spindles instead of bipolar spindles [19], [20], [21], [24], [57]. To assess the cellular phenotype of *Cenpj^tm/tm^* mice, MEFs were derived from *Cenpj* ^+/+^, *Cenpj* ^+/tm^ and *Cenpj^tm/tm^* littermates. For all experiments, MEFs were of early passage (P<5) and were passage-matched. Cenpj protein levels were reduced in the centrosomes of *Cenpj^tm/tm^* MEFs, but residual protein remained detectable in most cells (Figure 4A). Consistent with previous findings, frequencies of both monopolar and multipolar spindles were elevated in two independently derived *Cenpj^tm/tm^* MEF lines (Figure 4B; *Cenpj* ^+/+^: 2.1% monopolar and 8% multipolar; *Cenpj^tm/tm^* (1): 10.6% monopolar and 20.8% multipolar; *Cenpj^tm/tm^* (2): 8.9% monopolar and 18.9% multipolar). Distribution and intensities of the centrosomal proteins γ-tubulin (Figure 4A), CDK5RAP2 (Figure 4B), and pericentrin (data not shown) were unaffected in the mutant. We next asked whether spindle abnormalities are accompanied by aberrant centrosome and centriole numbers in *Cenpj^tm/tm^* MEFs. A normal mitotic cell contains two centrosomes, each containing a pair of centrioles. Supernumerary centrioles were visible in mitotic *Cenpj^tm/tm^* cells (Figure S5). To facilitate counting of centrioles, cells were arrested in mitosis using monastrol, a microtubule motor poison that prevents separation of spindle poles and thereby generates monopoles [58]. Cells with three or four centrioles were considered normal, since it is not always possible to resolve centrioles within a pair. We observed an increase in cells containing both too few (≤2) and too many centrioles (≥5) in the mutant (Figure 4C). To survive, cells with supernumerary centrosomes must either inactivate these or cluster active centrosomes into two poles, a process that ensures bipolar division [23], [59]. Clustered centrosomes were indeed observed in *Cenpj^tm/tm^* MEFs (Figure 4B). Centrosome clustering however does not prevent unequal partitioning of centrosomes into daughter cells (Figure S5), which ultimately causes a disassociation between centrosome numbers and DNA ploidy.

**Figure 5:**
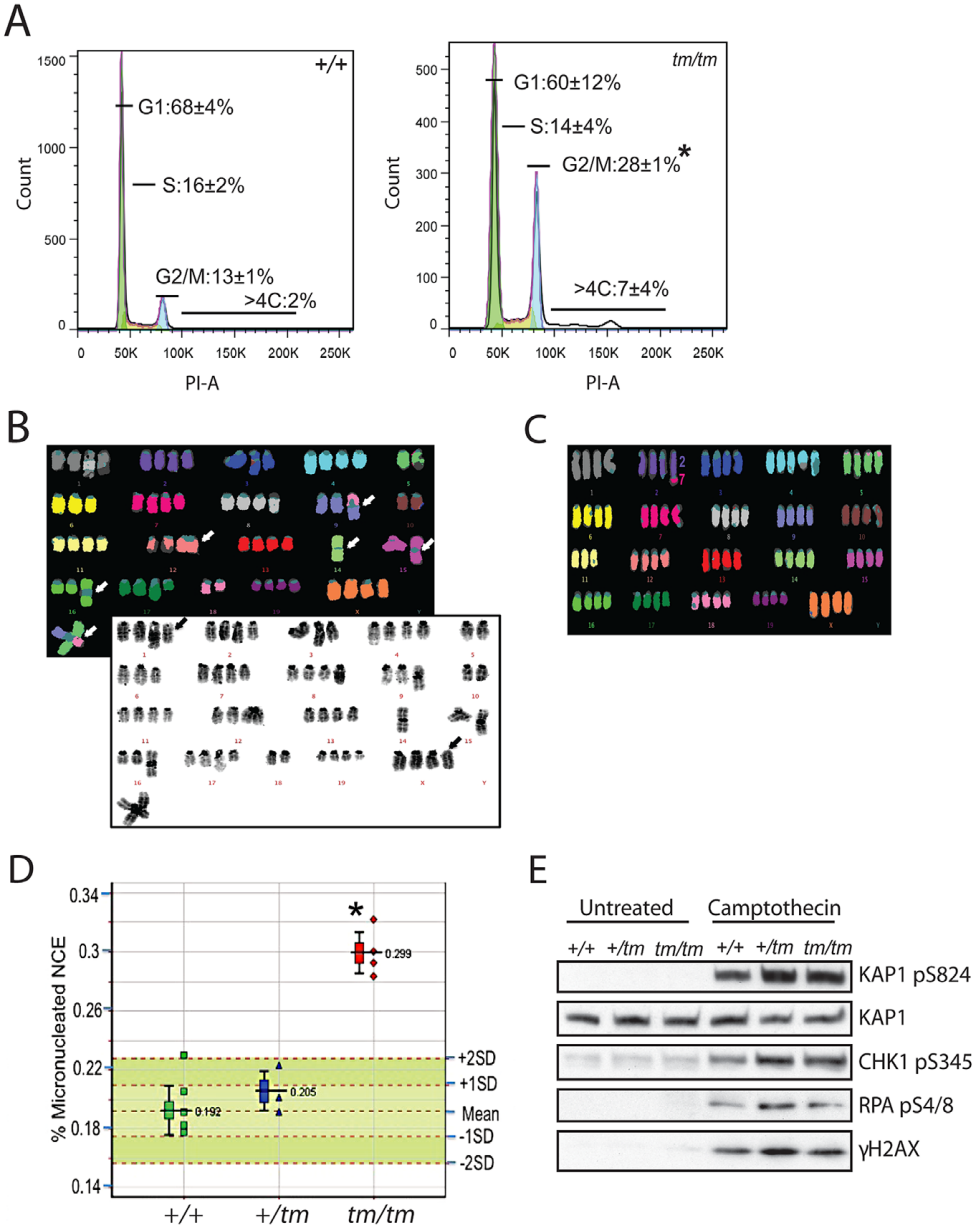
Genomic instability is associated with abnormal ploidy of *Cenpj^tm/tm^* cells rather than an impaired DNA damage response. A. Cell cycle analysis of *Cenpj^tm/tm^* mouse embryonic fibroblasts (MEFs) by flow cytometry showed an increase in the percentage of cells in G2 (4C) and cells containing >4C DNA content when compared to *Cenpj* ^+/+^ cells. Percentages represent means of n = 3 independent MEF lines per genotype (each pair of +/+ and *tm/tm* cells were passage-matched (passage<5) and derived from littermates), *P<0.05, t-test. PI, propidium iodide. B. Example multiplex fluorescent *in situ* hybridization (M-FISH; top) and DAPI banded (bottom) karyotype of a *Cenpj^tm/tm^* MEF metaphase (passage 4). The karyotype is near tetraploid, with centric fusions (white arrows) and chromosomes that have apparently lost their centromeres (black arrows). C. Example M-FISH of a *Cenpj^tm/tm^* MEF metaphase (passage 4) showing near tetraploid karyotype with a translocation (t(2;7)). D. Adult *Cenpj^tm/tm^* (n = 4) mice showed increased genomic instability when compared to *Cenpj* ^+/+^ mice (n = 6) as determined by the increased prevalence of micronucleated normochromatic erythrocytes using a flow cytometric assay of micronucleus formation. *P = 0.000004, t-test. The lower whisker extends to the lowest datum still within 1.5 Inter-quartile range (IQR) of the lower quartile. The upper whisker extends to the highest datum still within 1.5 IQR of the upper quartile. E. Immunoblots show normal activation of DNA damage response markers in *Cenpj*-deficient MEFs (passage 2) before and after treatment with the DNA damaging agent camptothecin (1 µM for 1 h). KAP1 was used as a loading control.

Cell-cycle analysis of *Cenpj^tm/tm^* MEFs revealed a significant increase in the number of 4C and elevated levels of >4C cells (Figure 5A), indicative of polyploidy. We examined the ploidy of fifty metaphase spreads of *Cenpj^tm/tm^* and *Cenpj* ^+/+^ MEFs and found that a remarkably high percentage of *Cenpj^tm/tm^* cells were near tetraploid (*Cenpj* ^+/+^ 11% vs. *Cenpj^tm/tm^* 41%). Twenty metaphase spreads with good fluorescent *in situ* hybridization (FISH) signals were selected for multiplex-FISH karyotyping which confirmed that many cells were near tetraploid and revealed additional defects such as aneuploidy, centromere loss, centric fusions (Figure 5B) and translocations (Figure 5C; for a breakdown of anomalies see Figure S2F). Consistently, we found evidence of lagging chromosomes in anaphase *Cenpj^tm/tm^* MEFs (Figure S5). Furthermore we show that adult *Cenpj^tm/tm^* mice have an increased prevalence of micronucleated normochromatic erythrocytes (*P* = 0.000004, t-test; Figure 5D), thus confirming that these mutants have spontaneous genomic instability.

### The genomic instability observed in *Cenpj^tm/tm^* mice does not reflect defective ATR- or ATM-dependent DNA–damage signaling

Seckel syndrome belongs to a group of genome instability disorders collectively referred to as DNA-damage response and repair-defective syndromes [60]. So far, all cells derived from Seckel patients have been found to be impaired in signaling mediated by the DNA-damage responsive protein kinase ATR, and therefore display reduced phosphorylation of downstream ATR substrates such as the checkpoint kinase Chk1, and have impaired G2/M cell-cycle checkpoint arrest upon treatment with DNA-damaging agents [60], [61]. We therefore treated fibroblasts from *Cenpj^tm/tm^* embryos (13.5 d.p.c) with the DNA damaging agent camptothecin, a DNA topoisomerase I inhibitor that causes DNA double-strand breaks specifically in S-phase [62]. Analyses revealed that *Cenpj^tm/tm^* fibroblasts were proficient for ATR-dependent and ATM-dependent phosphorylation of Chk1 (pS345) and KAP1 (pS824), respectively, and showed normal activation of γH2AX (Figure 5E). We found no evidence of an impaired G2/M DNA damage checkpoint as determined by the percentage of MPM2-positive cells following irradiation (Figure S2G). Furthermore, CtIP-Seckel cells show defective phosphorylation of replication protein A (RPA) after camptothecin treatment, a phenotype associated with impaired DNA-end resection and homologous recombination [6]. However, we found no evidence of this in *Cenpj^tm/tm^* MEFs (Figure 5E).

## Discussion

The *Cenpj* hypomorphic mouse (*Cenpj^tm/tm^*) that we have created displays many of the classical clinical features of Seckel syndrome, including intrauterine and postnatal dwarfism, microcephaly, a sloping forehead, neuropathogical abnormalities, memory impairment and genomic instability [1], [4], [7], [15], [28], [63]. In addition, we have shown that *Cenpj^tm/tm^* mice display some of the less frequently reported characteristics of the syndrome, including retarded bone ossification [1], [32], [64], as well as vertebral abnormalities and several other interesting histopathological and hematological abnormalities that have not previously been reported in patients.

### Microcephaly versus dwarfism of *Cenpj^tm/tm^* mice

Neuroepithelial cells have apical-basal polarity, and the switch from proliferative, symmetric to neurogenic, asymmetric division is controlled by the orientation of the spindle pole during mitotic division [65]. Primary microcephaly is caused by mutations of centrosomal proteins and is thought to arise from an increase in asymmetric divisions that reduces the size of the neural progenitor pool available for future brain growth, hence the growth deficit is restricted to the brain [66]. Seckel syndrome is characterized by microcephaly and a small body size (proportionate dwarfism). Interestingly, different mutations in the centrosomal proteins *CENPJ* or *CEP152* can cause microcephaly or Seckel syndrome [4], [14], [15]. The *CENPJ*-microcephaly mutations reported to date affect exons 2 (17delC), 11 (3243–3246delTCAG) and 16 (A3704T) [14], [15]; these mutations are predicted, but not proven, to cause defects in spindle pole orientation and proliferation of neural progenitors in a similar manner to other microcephaly genes. *CENPJ*-Seckel syndrome has been associated with a homozygous splice acceptor mutation in the last nucleotide of *CENPJ* intron 11 that results in the skipping of either exon 12, exons 12 and 13 or exons 11,12 and 13 during transcription [4]. This may represent a cellular attempt to salvage this important protein since the latter two transcripts are predicted to result in in-frame deletion and preservation of the C-terminus [4].

Notably, we found that insertion of a cassette between exons 4 and 5 of *Cenpj* resulted in splicing over the cassette and cryptic splicing, such that three different *Cenpj* mRNAs were expressed at very low levels: full length, one lacking exons 4 and 5 and one lacking exon 5. Skipping of exons 4 and 5 or exon 5 is predicted to result in a premature stop codon and protein products that are truncated after translation of exon 3 or 4, respectively. Immunoblotting and immunofluorescence revealed higher than expected levels of apparently full-length Cenpj protein were present in *Cenpj^tm/tm^* MEFs, which may be the result of post-transcriptional or translational regulation since the level of full-length *Cenpj* mRNA in *Cenpj^tm/tm^* MEFs was only 2% of wild-type levels. Furthermore, mRNA levels varied greatly between *Cenpj^tm/tm^* mouse embryonic fibroblasts, which may be due to genetic modifiers.

Together with studies of *CENPJ*-Seckel cells, which have shown that mutations in *CENPJ* may result in exon skipping and the generation of multiple transcripts that may generate in-frame protein products [4], these data suggest that expression of this critical protein may be rescued to some extent by cryptic splicing over deleterious mutations. These produce alternatively spliced mRNAs and thus the same mutation might not result in the same mRNA or protein levels in each individual. Moreover, cryptic splicing may also differ between tissues. Without a complete examination of the effects of different *CENPJ*-mutations on mRNA levels and splicing, and CENPJ protein levels, it is difficult to say why *CENPJ* mutations can either result in primary microcephaly or Seckel syndrome, or why the *Cenpj^tm1a(EUCOMM)Wtsi^* allele results in a mouse with a Seckel syndrome-like phenotype. However, we propose that *Cenpj^tm/tm^* mice display a Seckel syndrome-like phenotype, rather than primary microcephaly, due to a major reduction in full length Cenpj protein and therefore a lack of the protein domain(s) encoded by exons 11, 12 and/or 13.

The microcephaly of *Cenpj^tm/tm^* mice (brain weight two standard deviations below the mean) was not as severe as *CENPJ*-Seckel syndrome patients, who display anthropometric values that are all at least seven standard deviations below the mean [4]. The evolutionary lineage leading to humans is marked by a dramatic increase in brain size, suggesting that disruption of genes involved in neurogenesis will have a less profound effect in mice than in humans [67]. However, this is confounded by the fact that there are several mouse models of microcephaly, such as the humanized *ATR*-Seckel mouse and the *Cdk5rap2* mutant mouse, which display severe reductions in brain size [25], [37]. The discrepancy between microcephaly of *Cenpj^tm/tm^* mice and *CENPJ*-Seckel patients may instead be due to the hypomorphic nature of the *Cenpj^tm1a(EUCOMM)Wtsi^* allele or by the rapid evolution of the *Cenpj* gene between mice (80% sequence identity) and humans; the human CENPJ protein may be more efficient at regulating neurogenesis than that of the mouse [67].

The dwarfism and microcephaly of *Cenpj^tm/tm^* mice appeared to be the result of widespread DNA damage and apoptosis in embryos, rather than a reduction in cell proliferation. The level of cell death within the forebrain of the *Cenpj*-hypomorph embryonic mouse brain was comparable with that of the humanized ATR-Seckel mouse (approximately 1.5%; [25]). Similarly, *ATR*-, *CtIP*-, *CEP152*- and *PCNT*-Seckel cells have increased levels of DNA damage and a lowered apoptotic threshold with no change in the rate of proliferation [5], [6], [25]. In contrast to cells from *ATR*-, *CtIP*-, *CEP152*- and *PCNT*-Seckel syndrome patients, we have shown that MEFs from *Cenpj*-deficient mice are not impaired in ATR-dependent DNA damage signaling but instead show an elevated frequency of extra centrioles, multipolar spindles, and near tetraploid karyotypes. We suspect that the embryonic fibroblast line showing 41% near tetraploid cells could come from an embryo that would not have survived to term, indicating that genomic instability may also explain the sub-Mendelian birth ratio of *Cenpj^tm/tm^* mice. We also found evidence of chromosome missegregation, chromosomal translocations and centric fusions in *Cenpj^tm/tm^* MEFs. Increased levels of pan-nuclear γH2AX in embryos may be the result of chromosome breakage, micronucleus formation or missegregation [68], however it is possible that this reflects phosphorylation of H2AX during apoptosis-driven fragmentation of DNA [69].

### *Cenpj* is required for normal neuronal density and long-term memory

The neuropathological features of *Cenpj^tm/tm^* E14.5 embryos were remarkably similar to fetal stage Seckel syndrome. At E14.5, we found there was a reduction in neuron density within the developing telencephalon of *Cenpj^tm/tm^* mice. There are only two neuropathological reports of fetal stage Seckel syndrome (30 weeks gestation), although both showed that the cortical layers of the telencephalon were thin and that neuronal populations were less dense and less organized than age- or length-matched controls [28], [29]. As with *Cenpj^tm/tm^* mice, the hippocampal formation was short in one fetus, but displayed normal cytoarchitectural progression [28], [29]. Both reports indicated that the major nuclear groups of the basal ganglia, thalamus, cerebellum and brainstem showed no abnormalities in fetal stage Seckel syndrome [28], [29]. Interestingly, we saw a >50% reduction in the number of *Cenpj^tm/tm^* embryos between mid neurogenesis (E14.5) and the completion of neurogenesis (E18.5), when *Cenpj* is strongly expressed in the ventricular layers of the diencephalon, telencephalon, midbrain and cerebellum (www.emouseatlas.org, www.eurexpress.org), suggesting that *Cenpj*-deficiency during this critical period of neurogenesis causes partial lethality.

The majority of patients with Seckel syndrome are reported to have an IQ of <50 and are delayed in speech and reaching motor milestones, as well as displaying pyramidal signs, hyperactivity and an attention deficit [3], [34]. Cranial MRI of adult patients with Seckel syndrome has shown a reduction in brain volume, especially the cerebral cortex, a simplified gyral pattern (number of gyri reduced and shallow sulci), poorly developed frontal lobes, agenesis of the corpus callosum, reduction of white matter, brainstem and cerebellar hypoplasia, and dysmorphic or enlarged lateral ventricles [32], [34], [35]. A relatively normal MRI was reported for two siblings (aged two and four years-old) of the *CENPJ*-Seckel kindred and together with two cousins (aged five and six years-old), all had a history of normal cognitive and motor development [4]. The third cousin (MRI not performed, aged 16 years-old) had an IQ<60. Similarly, the brain regions of adult *Cenpj^tm/tm^* mice appeared anatomically proportionate, although these mice had a significantly shorter dentate gyrus than controls and this was accompanied by cognitive impairments reminiscent of Seckel syndrome patients.

### Centrioles, mitotic spindles, and ploidy

dSas-4 is the *Drosophila* homologue of CENPJ. Unlike dSas-4-depleted cells or dSas-4 mutant flies that progressively lose centrioles, *Cenpj^tm/tm^* MEFs contain centrioles even after several passages [70], [71]. While the increase in *Cenpj^tm/tm^* cells with two or fewer centrioles is consistent with an impairment of centriole assembly, this effect is relatively mild, and therefore suggests that the mutant expresses residual, functional Cenpj protein. Ciliogenesis requires centriole biogenesis and therefore dSas-4 mutants lack both primary and motile cilia [70]. The role of CENPJ in ciliogenesis has not been extensively explored in mammals, but depletion of CENPJ in cultured cells is reported to impair primary cilium formation [72]. *Cenpj^tm/tm^* mice (16 weeks old) did not display phenotypes normally associated with ciliopathies such as *situs inversus* or renal cystic disease, suggesting that sufficient amounts of Cenpj are available in the mutant for cilia formation in the majority of cells. However, the abnormalities in ciliary processes and photoreceptor nuclei within the eye may be attributed to ciliary defects. Moreover, unlike dSas-4 mutant males that display loss of flagella and sperm motility, *Cenpj^tm/tm^* male mice are fertile [70], which could again be due to residual expression of *Cenpj*.

While *Cenpj^tm/tm^* MEFs displayed irregular centriole numbers and mono- and multipolar spindles, they also showed extensive polyploidy and aneuploidy. Thus, we cannot conclude whether abnormal centriole and centrosome numbers are the cause or consequence of aberrant ploidy. Figure S6A shows the possible sequence of events that may lead to the abnormal ploidy of *CENPJ*-Seckel cells. Aberrant centrosome numbers are known to cause mitotic spindle abnormalities, culminating in mitotic delay, chromosome missegegration, cytokinetic failure and polyploidy. Prolonged mitotic delay can cause DNA damage, cell cycle arrest and apoptosis [73], [74]. Chromosome missegregation can also damage chromosomes, hence triggering activation of DNA damage checkpoints [68], [75]. Chromosome instability could therefore explain the increase in γH2AX levels and potentially, the increase in apoptosis in the mutant embryonic brain. Of all chromosome aberrations detected in the mutant MEFs, tetraploidy was the most prominent. A common cause of tetraploidy is an abortive mitotic cell cycle whereby cells enter but fail to complete mitosis [76]. Mitotic spindle abnormalities in *Cenpj^tm/tm^* cells could trigger extended mitotic arrest followed by mitotic slippage producing a tetraploid cell (Figure S6A). Tetraploid *Cenpj^tm/tm^* MEFs seem to be able to proliferate, since they represented almost 40% of the metaphase cells obtained for karyotyping. Interestingly, dSas-4 mutant flies show only a small increase in the proportion of aneuploid cells (1% in wild-type vs. 3% in mutants) and no polyploidy [70], whereas the proportion of near tetraploid *Cenpj^tm/tm^* embryonic fibroblasts was surprisingly high (∼10% in wildtype vs ∼40% in *Cenpj^tm/tm^* MEFs). We suspect that *Cenpj*-deficiency exacerbates tetraploidy in MEFs, which are particularly susceptible to tetraploidy with passaging [77]. Nonetheless, adult *Cenpj^tm/tm^* mice show increased micronucleus induction, which is likely the result of lagging chromosomes and chromosome breakage.

### Polyploidy as a potential cause of karyomegaly in *Cenpj^tm/tm^* tissues

*Cenpj^tm/tm^* mice of both genders showed an increased incidence of hypertrophic, disorganized cardiomyoctes with karyomegaly in the endocarium and interventricular septum when compared to wildtype mice. The areas showed no evidence of degeneration or repair, however since a high proportion of *Cenpj^tm/tm^* MEFs are polyploid, this is likely to be the cause of the karyomegaly. Although one of the less frequently reported characteristics of Seckel syndrome, there are numerous case-reports of severe cardiac anomalies in Seckel syndrome patients, including atrial and ventricular septal defects, pulmonary atresia, patent ductus arteriosus and congenital heart disease [49], [50], [51], [52], [53]. It will be interesting to see whether *CENPJ*-Seckel patients develop cardiac defects as they age. At 16 weeks of age *Cenpj^tm/tm^* mice showed hypoalbuminemia, which is associated with chronic liver and kidney diseases, although histopathological analysis of their livers and kidneys did not reveal any abnormalities. However, the preponderance of karyomegaly in the liver and Harderian glands was increased in aged *Cenpj^tm/tm^* mice. Cenpj-deficiency may exacerbate this phenomenon in the cells of both of these tissues, which are prone to karyomegaly [78].

### Susceptibility to malignancy

Familial syndromes associated with genomic instability often predispose to cancer formation since DNA damage is the source of mutations that drive malignant transformation. However only a few cancers have been reported for Seckel syndrome patients, possibly due to the shorter life-expectancy of patients with primordial dwarfism. Furthermore, since mutation of each of the five known Seckel genes, *ATR, PCNT, CENPJ, CEP152* and *RBBP8 (CtIP)*, cause genomic instability that is associated with apoptosis, it is possible that Seckel cells may not have the opportunity to accumulate cancer-causing mutations. The chromosomal instability that is associated with *Cenpj*-deficiency could result in aneuploidy or translocations that cause loss of tumour suppressors or the formation of oncogenic fusion proteins, respectively [79]. We are currently ageing a cohort of *Cenpj^tm/tm^* mice (currently 7–14 months old) to determine whether *Cenpj*-deficiency alters the frequency of malignancy or shortens life-expectancy.

### *Cenpj*-deficient mouse phenotypes for which there are currently no clinical correlates

We noted a small number of abnormalities in *Cenpj^tm/tm^* mice that have not been previously reported for Seckel syndrome patients or mouse models. Seckel syndrome is associated with ocular defects in humans, including spontaneous lens dislocation, myopia, astigmatism, and retinal degeneration. Ocular examination of *CENPJ*-Seckel patients has not yet been reported [47], [48], however Cenpj was highly expressed in the rapidly proliferating retinal neuroblast layer in the 14.5 d.p.c. mouse embryo and *Cenpj*-deficient mice presented with a number of ocular abnormalities. Furthermore, a small number of reports suggest that Seckel-like syndromes are associated with precocious puberty or premature thelarche [44], [45]. In contrast, female *Cenpj^tm/tm^* mice showed signs of delayed puberty, although the reproductive tract appeared normal at 16 weeks. We built a protein-protein interaction network using all known Seckel Syndrome associated genes as query (Figure S6B). By using gene ontology enrichment analysis we showed that 265 biological processes (level 3 classification) are significantly over-represented in the Seckel syndrome network (Table S1). As expected, many of the processes were involved in the regulation of cell cycle, cell growth and cell death. Interestingly, the network was also enriched for genes involved in the ‘response to hormone stimulus’ ‘ovulation cycle process’ and ‘sex differentiation’ (Table S1). Transient insulin resistance during puberty is a well documented phenomenon [80]. *Cenpj^tm/tm^* mice of both genders had a delayed response to glucose challenge although this was more marked in 16 week-old female mice, which may be explained by delayed puberty in female *Cenpj^tm/tm^* mice. While there are no reports of an association between abnormal glucose homeostasis and Seckel syndrome, interestingly, most individuals with MOPDII, including *PCNT*-MOPDII, develop insulin resistance and diabetes during childhood [81], [82]. Aside from centrosome-mediated regulation of the cell-cycle, PCNT is thought to regulate insulin secretory vesicle docking in mouse pancreatic β-cells [83]. Whether CENPJ plays a role in glucose homeostasis remains to be determined. Finally, the proportion of CD8+CD3+ T cells were elevated in *Cenpj^tm/tm^* mice, although this was more pronounced in males. The Seckel syndrome protein-protein interaction network that we generated was significantly enriched for genes involved in ‘leukocyte mediated immunity’, ‘leukocyte mediated cytotoxicity’, ‘leukocyte activation’ and ‘interleukin-2 production’ (Table S1). While we are uncertain of the biological basis for these relationships, it will be interesting to see whether there are clinical correlates for these abnormalities and whether other mouse models of Seckel syndrome or primordial dwarfism share these anomalies.

### Summary

Mouse models of Seckel syndrome may go some way towards the molecular genetic delineation of this heterogeneous condition. The generalized activation of apoptosis as a result of genomic instability in *ATR*-Seckel and *Cenpj^tm/tm^* mouse embryos provides one explanation for the proportionate dwarfism of Seckel syndrome patients. In agreement with the intron 11 *CENPJ*-Seckel mutation, which results in the formation of three transcripts, we showed that *Cenpj* expression is rescued to some extent by cryptic splicing over the cassette to produce a variety of truncated mRNAs, and that there is a moderate degree of individual variation in the ability of an organism to perform this rescue. These data highlight the need for detailed mRNA expression, splicing studies, and protein analysis to establish how individual mutations affect the normal and cryptic splicing of *CENPJ* mRNAs for each patient directly, and not with prediction analysis tools, so as to understand why some *CENPJ* mutations cause microcephaly and others Seckel syndrome and how the same *CENPJ* mutation can cause clinical heterogeneity [4].

## Materials and Methods

### Confirmation of correct targeting and animal husbandry

Mutant mice carrying the *Cenpj^tm1a(EUCOMM)Wtsi^* allele were generated on a C57BL/6NTac; C57BL/6-Tyr^c-Brd^ background (clone EPD0028_7_G05) by the Sanger Mouse Genetics Project as part of the European Conditional Mouse Mutagenesis Program (EUCOMM; [26]). Correct gene targeting in founder mice was determined by a combination of standard PCR and quantitative PCR (qPCR; see Figure S1 for more details). Following confirmation of correct targeting, mice were genotyped for the *Cenpj^tm1a(EUCOMM)Wtsi^* allele by PCR using primers specific to the wildtype and mutant *Cenpj* alleles and to LacZ (Figure S1D). In individual experiments, all mice were matched for age and gender. A cumulative baseline was generated from data arising from controls from the same genetic background, age and gender. The care and use of all mice in this study was carried out in accordance with UK Home Office regulations, UK Animals (Scientific Procedures) Act of 1986. Mice were maintained in a specific pathogen free unit on a 12 hr light: 12 hr dark cycle with lights off at 7:30pm and no twilight period. The ambient temperature was 21±2°C and the humidity was 55±10%. Mice were housed using a stocking density of 3–5 mice per cage (overall dimensions of caging: (L×W×H) 365×207×140 mm, floor area 530 cm^2^) in individually ventilated caging (Tecniplast Seal Safe1284L) receiving 60 air changes per hour. In addition to Aspen bedding substrate, standard environmental enrichment of two nestlets, a cardboard Fun Tunnel and three wooden chew blocks was provided. Mice were given water and diet *ad libitum*. At 4 weeks of age, mice were transferred from Mouse Breeders Diet (Lab Diets, 5021–3) to a high fat (21.4% fat by crude content) dietary challenge (Special Diet Services, Western RD 829100).

### Quantitative real-time PCR

To test for expression of *Cenpj* and cryptic splicing, total RNA was isolated from mouse embryonic fibroblasts (13.5 days post coitum (d.p.c.), n = 3) using the RNeasy minikit (Qiagen). RT-PCR was performed using the BD Sprint kit containing random hexamers (BD Clontech, CA, USA). Primers were designed to exon boundaries and sequences are available on request. cDNA was quantified using SYBR Green on an ABI7900HT (ABI, CA, USA). Gene expression was normalised to *Gapdh* and to wild-type control.

### Immunoblotting

Protein extracts were prepared from mouse embryonic fibroblasts (13.5 d.p.c.) by directly harvesting cells in Laemmli buffer. Proteins were separated by SDS-PAGE, and membranes were incubated with primary antibodies to CENPJ (Stratech, Newmarket, UK. Rabbit 1∶500), KAP1 (Abcam, Cambridge, UK. Rabbit 1∶10 000), KAP1 phospho-Ser-824 (Bethyl. Texas, US. Rabbit 1∶1000), Chk1 phospho-Ser-345 (Cell Signaling, Boston, US. Rabbit 1∶5000), RPA phospho-Ser-4/8 (Bethyl, Texas, US. Rabbit 1∶10 000), H2AX phospho-Ser-139 (γH2AX; Millipore, Billerica, US. Mouse 1∶1000). Camptothecin was from Sigma (Poole, UK).

### LacZ staining

LacZ staining was performed on tissues perfused, removed and fixed (30 min) with 4% paraformaldehyde (pH8). Tissues were then washed three times in PBS. Tissues were then incubated at 4°C for 48 h in staining buffer (5 mM K_3_Fe(CN)_6_, 5 mM K_4_Fe(CN)_6_, 0.02% IGEPAL CA-630, 0.01% deoxycholate, 2 mM MgCl_2_, 1 mg/ml bromo-chloro-indolyl-galactopyranoside in the dark. Tissues were fixed again with 4% paraformaldehyde overnight at 4°C before being transferred through increasing glycerol concentrations and archiving in 70% glycerol+0.01% sodium azide.

### E18.5 measurements and skeletal preparations

At 18.5 d.p.c. litters were harvested and euthanized. The crown-rump length was measured and measurements of skulls were adapted from published methods used to assess adult mouse skulls [84]. Embryos were scalded at 67°C for 30 seconds to facilitate removal of skin, muscle and fat. Dissected embryos were then fixed in 100% ethanol for 48 h and transferred to acetone for 48 h to further de-fat the skeletons. Skeletal embryos were then stained with 0.015% Alcian Blue (Sigma-Aldrich, UK; in Ethanol/Glacial Acetic Acid) for 24 h, washed five times in 100% ethanol and transferred to 0.1% potassium hydroxide overnight. Skeletons were then stained with 0.005% Alizarin Red (Merck, UK; in 1% KOH) for 3 h, washed with 1% KOH three times and transferred to 20% Glycerol/1% KOH for clearing for ∼3 days. Skeletons were then transferred through increasing glycerol concentrations and archived in 70% glycerol +0.01% sodium azide.

### X-ray imaging

X-ray imaging was performed at 14 weeks of age using a Faxitron MX-20 cabinet (Faxitron Bioptics, IL, USA). Mice were anesthetized with a preparation of 100 mg/kg Ketamine/10 mg/kg Xylazine. Weight and body length were measured prior to the scan. Five images were acquired: dorsoventral and lateral images of the whole body at ×1 magnification, dorsoventral and lateral images of the head at ×4 magnification and a dorsoventral image of the left forepaw at ×5 magnification. Images were acquired using an energy of 23 kV for 10 seconds per image. Images were studied visually to assess abnormalities within 41 standard parameters. To show that humeri were anatomically disproportionate the right greater tubercle - deltoid tuberosity length (mm) was normalized to the right greater tubercle – trochlea length (mm) and data were represented as a percentage.

### Histology

Adult tissue samples were fixed in 10% neutral buffered formalin and E14.5 embryos were fixed in 4% PFA. Samples were dehydrated, paraffin embedded and 4 µm sections were cut before hematoxylin and eosin staining. Neuron densities of E14.5 embryo brains were determined by counting the number of nuclei in three different areas that were 75 µm^2^ for the densely populated ventricular zones and 150 µm^2^ for the less densely populated mid-striatum. Adult neuroanatomical measurements (Figure S3) were carried out on 40 µm thick Nissl stained sections using ImageJ freeware (NIH). Heart fibrosis was assessed by Masson's Trichrome and X-zone pigmentation of the adrenals was confirmed by Periodic Acid-Schiff's staining using standard methods. Whole mouse eyes were enucleated, fixed, and sectioned for histological studies as previously described [85].

### Immunohistochemistry

Sections of paraffin embedded tissues were dewaxed and antigen retrieval was performed in boiling 10 mM citrate buffer pH6. Endogenous peroxidases were quenched in 3% hydrogen peroxide (Sigma, UK) before blocking in normal serum (VectorLabs, UK). Sections were incubated in primary antibodies to Cenpj (Stratech), cleaved caspase-3 (Cell Signaling Technologies, CA, USA), Ki67 (DAKO Ltd, UK) and phospho-Ser 139 H2AX (γH2AX, Cell Signaling Technologies, CA, USA). Vectorstain ABC kit and DAB (VectorLabs, UK) were used according to the manufacturer's instructions. Sections were counterstained with haematoxylin before clearing and mounting. The number of cells positive for cleaved-caspase 3 or γH2AX were counted in two areas of 75 µm^2^ the striatum, cortex and pro-hippocampus and data shown are the percentage of total cells in the area. Counts were performed independently by two individuals to confirm reproducibility.

### Neurobehavioral and sensory assessment

At nine weeks of age, open field, grip strength and modified SHIRPA were performed as described previously [86], [87]. Hot plate assessment was performed at 10 weeks of age using standard techniques. Tests for social recognition and olfaction (*Cenpj^tm/tm^* n = 9 and *Cenpj* ^+/+^ n = 8) were performed on male mice at 3–6 months of age. Mice were habituated to a test arena identical to their home cage for 10 min. For social recognition, a stimulus mouse was placed into the test arena for 1 min, repeated four times at 10 min intervals. In the fifth trial, a second stimulus mouse was presented. 24 h later the test animals were presented with the familiar animal from trials 1–4 and a new unfamiliar animal, for 2 min. Trials were performed under red light and recorded with an overhead camera and the videos scored blind of genotype. The amount of time the test animal spent investigating, by oronasal contact or approaching within 1–2 cm, was recorded. Stimulus animals, 2–4 months old, were weight-matched to *Cenpj^tm/tm^* mice and sedated with ketamine/xylazine (i.p. 1 g/0.1 g per kg of body weight). C57BL/6NTac mice were used for trials 1–4 and for the 24 h discrimination test, 129P2/OlaHsd mice were used for trial 5. Three animals (two *Cenpj^tm/tm^* and one *Cenpj* ^+/+^) were taken out of the analysis because of their low investigation times (less than 10 s on trial one or during the discrimination test).

### Glucose tolerance

We assessed glucose tolerance in mice fed on a high-fat diet (Western RD, 829100, Special Diets Services) from 4 weeks of age until 13 weeks of age. At 13 weeks, mice were fasted overnight before a blood sample was taken and glucose was measured using an Accu-Chek Aviva (Roche). To perform an intra-peritoneal glucose tolerance test (IP-GTT), mice were fasted for 16 h, a bolus of glucose was administered intraperitoneally and blood glucose concentration from the tail vein was measured using Accu-Chek Aviva (Roche) after 15, 30, 60 and 120 min.

### Clinical chemistry

We performed clinical chemistry on 16-week-old mice. Mice were terminally anaesthetized and blood was collected from the retro-orbital sinus into lithium-heparin tubes. The plasma was immediately analyzed on an Olympus AU400 Analyzer.

### Flow cytometry

At 16 weeks of age blood samples were collected into heparin-coated tubes. The main leukocyte populations in peripheral blood were characterized by 8-colour flow cytometry with an LSRII (BD Bioscience, UK Biosciences, UK) and associated software. Briefly, samples were centrifuged at 5000 *g* for 10 min at 8°C to remove the plasma layer. Red blood cells were lysed (Pharmalyse, BD Bioscience, UK Biosciences, UK) and samples were centrifuged at 400 *g* for 3 min to pellet the white cells. White cells were resuspended in buffer (PBS pH 7.45 containing 0.5% BSA), transferred to 96-well plate and washed in buffer several times before incubation in 50 µl 10 µg/ml Mouse FcBlock (BD Bioscience, UK Biosciences, UK) for 15 min on ice. Cells were washed in buffer and incubated with 50 µl antibody mix from two staining panels (Tables S2 and S3) for 15 min on ice. Propidium iodide (2.5 mg/ml; Sigma, UK) was added to each well and cells were incubated for a further 5 min. Cells were washed in buffer several times before 30 000 propidium iodide-negative, CD45-positive events were collected. Data were interpreted using FlowJo (v7.6, Tree Star, Inc., OR, USA). The frequency of micronucleated normochromatic erythrocytes was determined by flow cytometry (FC500, Beckman Coulter, USA) as described previously [88] and data were interpreted using FlowJo (v9.3.1, Tree Star, Inc., OR, USA). Cell cycle analysis was performed on mouse embryonic fibroblasts (13.5 d.p.c.). Briefly, cells were fixed in 70% ethanol overnight and stained with PI solution before analyzing on a flow cytometer (LSR Fortessa, BD, USA). A total of 10,000 events were acquired per sample. The cells were gated on PI fluorescence area versus PI fluorescence width to discriminate any doublets and clumps. The gated events were displayed on a histogram plot of PI fluorescence area. Data were analyzed using FlowJo (v9.3.1, Tree Star, Inc., OR, USA).

### Immunofluorescence

Mouse embryonic fibroblasts (13.5 d.p.c.) were collected and cultured using standard methods. Primary antibodies used in this study were CDK5RAP2 (Bethyl), centrin-3 (Abnova), TPX2 (Abnova), gamma-tubulin (GTU88; Sigma-Aldrich), alpha-tubulin (DM1A; Sigma-Aldrich). Secondary antibodies conjugated to Alexa Fluor 488 and 555 (Invitrogen) were used. DNA was stained with Hoescht (Sigma-Aldrich). To detect centrosomal markers, cells were fixed in −20°C methanol for 5 min. After fixation, cells were processed for immunofluorescence and microscopy as described in [89]. For centriole counts in Figure 4C cells were treated with 100 µM monastrol for 16 h before fixation.

### G2 checkpoint assay

Cells were irradiated with 3 Gy ionizing radiation using a Faxitron cabinet X-ray system and left recover for 8 h in the presence of 1 µg/ml of nocodazole (Sigma, Poole, UK) to trap mitotic cells. Cells were trypsinized and fixed with 4% paraformaldehyde, permeabilized with 1× phosphate buffered saline containing 0.2% Triton X-100 for 30 min on ice, and incubated in the presence of MPM2 primary antibodies (Millipore, Billerica, US. Mouse 1∶100) and then Alexa-Fluor-488 secondary antibodies (Life Technologies, Paisley, Scotland. Goat anti-mouse 1∶200) to detect mitotic cells.

### Multiplex-fluorescence *in situ* hybridization (FISH)

Metaphase spreads from MEFs (*Cenpj^tm/tm^* and *Cenpj^+/+^* littermates) were prepared as for metaphase FISH and multiplex-FISH was carried out as previously described [90].

### Network generation and analysis

A network of Protein-Protein interactions for Seckel syndrome genes was generated using the Cytoscape 2.8.1 [91] plug-in BisoGenet 1.41.00 [92]. The Entrez Gene symbols of the Seckel syndrome associated genes (*CENPJ, PCNT, CEP152, ATR, RBBP8 (CtIP), SCKL3*, Entrez Gene IDs = 55835, 5116, 22995, 545, 5932 and 386616 respectively) [4], [5], [6], [41] were used as query to build a network of experimentally validated Protein-Protein interactions, by adding neighbours to the input nodes up to a distance of one. All the interactions are downloaded by BisoGenet from its own database SysBiomics and have been validated by one or different experimental methodologies such as X-ray crystallography, surface plasmon resonance, two hybrid systems, three hybrid systems and Western blot. The network's characteristic path length was calculated using Cytoscape's built-in plug-in NetworkAnalyzer [93].

### Gene Ontology (GO) over-representation analysis

The GOs over-representation analysis was performed using the Over-representation analysis tool of the Consensus Path Database website [94]. Statistical significance of the different GOs represented in our network was calculated by the website's tool using a Hypergeometric test. After calculating the p-value, the tool corrects it for the false discovery rate generating the corrected q-value. A q-value <0.01 was used as threshold for all significant results.

### Statistical analyses

A Shapiro-Wilk normality test was performed to assess whether data were normally distributed followed by an F-Test to assess whether equality of variances could be assumed. The significance of the difference between the means of both data sets was tested by applying a two-sided T-Test, assuming variance equality whenever the F-Test was positive (*P*>0.05). For all cases where the Shapiro-Wilk normality test was negative (*P*>0.05) a Mann-Whitney non-parametric test was applied to assess the significance. Statistical analyses were performed using R-2.13.0 [95].

## Supporting Information

Figure S1 A. Design and validation of the *Cenpj* allele. The L1L2_gt1 cassette was inserted at basepair 57174548 of chromosome 14 upstream of a *Cenpj* critical exon (exon 5, Build 37). The cassette is composed of an FRT-flanked lacZ/neomycin sequence followed by a loxP site. An additional loxP site is inserted downstream of the targeted exon at basepair 57173663. The critical exon is thus flanked by loxP sites. Further information on targeting strategies used for this and other KOMP alleles can be found at http://www.knockoutmouse.org/aboutkompstrategies. B. Correct targeting in founder mice was confirmed by standard PCR using the primers shown in Figure S1D (for more details on cassette quality control see http://www.knockoutmouse.org/kb/entry/90/). Gels show the presence of LacZ, 5′FRT and LoxP sites, generation of a mutant band (MUT) and absence of backbone (VF4). C. Correct targeting was also confirmed by loss of wildtype allele qPCR. A TaqMan qPCR assay was designed to the wildtype sequence removed during recombineering of the mutant allele. Samples were amplified in a multiplex reaction with a Tfrc endogenous VIC labeled control (Applied Biosystems) and then compared to known wildtype controls using the ΔΔCt method. Loss of one copy in heterozygotes and no amplification at all in homozygotes strongly suggests that the targeting is correct. No loss in copy number would indicate either a wildtype mouse (confirmed by neo count qPCR) or an incorrect targeting event. Targeting was also confirmed by traditional end point PCR by a failure in homozygotes (detected by neo count qPCR) to amplify a product designed to the wild-type allele, using primers flanking the cassette insertion point. D. Primers used for quality control and genotyping.
(PDF)

Figure S2 Analyses of *Cenpj^tm/tm^* mice A. LacZ staining in the brain was restricted to the lining of the cerebellar aqueduct and the fourth ventricle, which connected to a focus of staining at the precommissural nucleus. Weak staining was observed around the ventromedial preoptic nucleus. Strong LacZ staining was observed in the testes (the epididymus contains endogenous staining) and moderate staining in the medulla of the kidneys (the cortex contains background staining). B. Cryptic splicing of *Cenpj*. Percentages show Mean±S.E.M. expression of *Cenpj* across exon boundaries as determined by quantitative RT-PCR relative to *Gapdh* for *Cenpj^tm/tm^* relative to *Cenpj^+/+^* for RNA extracted from n = 3 MEF lines. C. *Cenpj* coding begins in Exon 2. Blue font indicates alternate exons. Red font indicates amino acids encoded across a splice junction. Alternatively spliced transcripts (Ex3–6 and Ex4–6) result in truncated protein products. Yellow highlight indicates the point at which the protein goes out-of-frame resulting in a stop codon. D. Measurements of nose-to-tail base length of male *Cenpj^+/+^* (n = 37), *Cenpj^+/tm^* (n = 7), *Cenpj^tm/tm^* (n = 8) and baseline wild-type control (n = 790) mice at 14 weeks of age. Data show that male *Cenpj^tm/tm^* mice are significantly shorter than *Cenpj^+/+^* mice (*P = 2.2×10^−16^, t-test). The lower whisker extends to the lowest datum still within 1.5 Inter-quartile range (IQR) of the lower quartile. The upper whisker extends to the highest datum still within 1.5 IQR of the upper quartile. E. Representative haematoxylin and eosin stained sections. Karyomegaly (arrow heads) of cardiomyocytes was increased at 16 weeks of age in 5/6 *Cenpj^tm/tm^* and 1/6 *Cenpj^+/tm^* mice when compared to wild-type control (0/4; *Cenpj^tm/tm^* vs *Cenpj^+/+^*, *P* = 0.048, Fisher exact test). Histopathology at 13 months of age revealed an increased prevalence of karyomegaly in cardiomyocytes (17.6% (16/91) vs 3.3% (4/120)), hepatocytes (9.3% (10/108) vs 3.0% (3/101)) and cells of the Harderian glands (14.5% (18/124) vs 0.6% (1/155)) of *Cenpj^tm/tm^* vs. age-matched wild-type mice. Scale bars 25 µm. F. Twenty metaphases from *Cenpj^tm/tm^* and *Cenpj^+/+^* MEFs (passage 4, derived from littermates) were examined by multiplex fluorescent *in situ* hybridization. Table shows breakdown of numerical and structural chromosomal aberrations. G. Percentage of MPM2-positive MEFs (passage 2) following irradiation shows that the G2/M checkpoint is not impaired by *Cenpj*-deficiency.
(PDF)

Figure S3 Brain measurement analysis. Diagram to show the measurements of adult brains taken at 16 weeks of age. Thalamus, mammillothalamic tracts and caudate were used as markers.
(PDF)

Figure S4 Apoptotic cells are scattered throughout *Cenpj^tm/tm^* embryos. Representative whole embryo (14.5 d.p.c.) images show immunohistochemical staining for Cenpj, Ki67 as a marker of proliferation, cleaved (activated) caspase-3 as a marker of apoptosis and Ser139-phosphorylated H2AX (γH2AX) as a marker of DNA damage. Apoptotic cells were scattered throughout embryos and this was more apparent in the forebrain (fb), eyes and limbs. The pattern of γH2AX-positive staining was similar to cleaved caspase-3 however this was also more apparent in the trigeminal ganglion (tg) and liver. Scale bar 500 µm.
(PDF)

Figure S5 Centrosomal abnormalities of *Cenpj^tm/tm^* cells. A. Images show examples of centrin-3 staining in centrosomes of *Cenpj^+/+^* and *Cenpj^tm/tm^* mouse embryonic fibroblasts (MEFs). Cells were stained with antibodies against centrin-3 (red in merge) and the mitotic spindle protein TPX-2 (green in merge). DNA is in blue. Framed areas are shown at higher magnification. In *Cenpj^tm/tm^* MEFs several centrioles are clustered in a broad spindle pole. In the centre of the spindle two centrioles are visible: these do not associate with a pole and do not seem to nucleate a major microtubule aster, suggesting that these might be part of an inactive centrosome. B. An example for multiple lagging chromosomes in a cell with supernumerary centrosomes. Cells were stained with antibodies against the centrosomal protein CDK5RAP2 (red in merge). DNA is in green. Arrows mark lagging chromosomes. Scale bars are 5 µm.
(PDF)

Figure S6 Proposed mechanism of cell death of *CENPJ-SECKEL* cells and SECKEL protein interaction network. A. Flow diagram to illustrate the sequence of events that may lead to chromosomal instability, polyploidy or cell death of *CENPJ*-SECKEL cells. Aberrant or supernumerary centrosomes are likely to increase the frequency of multipolar spindle cell intermediates. Clustering centrosomes may enable a bipolar division, however the presence of extra centrosomes increases the frequency of merotelic microtubule-kinetochore attachment errors and leads to lagging chromosomes or missegregation of sister chromatids (aneuploidy). Alternatively, cells with supernumerary centrosomes may undergo a multipolar division; completion of cytokinesis would likely result in non-viable progeny, whereas failure of cytokinesis could result in tetraploidy. It is thought that aneuploid cells may also arise through tetraploid intermediates. B. Using all known Seckel Syndrome associated genes as query, we built a network with 130 genes (nodes) and 665 edges, where the edges represent an experimentally validated protein-protein interaction between the gene products. The pink colored nodes are the known Seckel –syndrome associated genes and the blue colored genes are the ones added by the network expansion analysis.
(PDF)

Table S1 Protein interaction network. Using gene ontology enrichment analysis of the Seckel syndrome protein-protein interaction network. 265 biological processes (level 3 classification) are over-represented and many are related to the regulation of the cell cycle, cell growth and cell death (q-val<0.01).
(XLSX)

Table S2 Peripheral blood leukocyte analyses: Staining panel 1. A list of antibodies use in the peripheral blood straining 1, the dilution they were used at, and the suppliers of these antibodies.
(DOCX)

Table S3 Peripheral blood leukocyte analyses: Staining panel 2. A list of antibodies use in the peripheral blood straining 2, the dilution they were used at, and the suppliers of these antibodies.
(DOCX)

## References

[1] Majewski F, Goecke T (1982) Studies of microcephalic primordial dwarfism I: approach to a delineation of the Seckel syndrome. American journal of medical genetics 12: 7–21. 7046443 10.1002/ajmg.1320120103

[2] Faivre L, Le Merrer M, Lyonnet S, Plauchu H, Dagoneau N, et al (2002) Clinical and genetic heterogeneity of Seckel syndrome. American journal of medical genetics 112: 379–383. 12376940 10.1002/ajmg.10677

[3] Harsha Vardhan BG, Muthu MS, Saraswathi K, Koteeswaran D (2007) Bird-headed dwarf of Seckel. Journal of the Indian Society of Pedodontics and Preventive Dentistry 25 Suppl: S8–9. 17921644

[4] Al-Dosari MS, Shaheen R, Colak D, Alkuraya FS (2010) Novel CENPJ mutation causes Seckel syndrome. J Med Genet 47: 411–414. 20522431 10.1136/jmg.2009.076646

[5] Kalay E, Yigit G, Aslan Y, Brown KE, Pohl E, et al (2011) CEP152 is a genome maintenance protein disrupted in Seckel syndrome. Nature genetics 43: 23–26. 21131973 10.1038/ng.725 PMC3430850

[6] Qvist P, Huertas P, Jimeno S, Nyegaard M, Hassan MJ, et al (2011) CtIP Mutations Cause Seckel and Jawad Syndromes. PLoS genetics 7: e1002310. 21998596 10.1371/journal.pgen.1002310 PMC3188555

[7] O'Driscoll M, Ruiz-Perez VL, Woods CG, Jeggo PA, Goodship JA (2003) A splicing mutation affecting expression of ataxia-telangiectasia and Rad3-related protein (ATR) results in Seckel syndrome. Nature genetics 33: 497–501. 12640452 10.1038/ng1129

[8] Majewski F, Ranke M, Schinzel A (1982) Studies of microcephalic primordial dwarfism II: the osteodysplastic type II of primordial dwarfism. American journal of medical genetics 12: 23–35. 7201238 10.1002/ajmg.1320120104

[9] Rauch A, Thiel CT, Schindler D, Wick U, Crow YJ, et al (2008) Mutations in the pericentrin (PCNT) gene cause primordial dwarfism. Science 319: 816–819. 18174396 10.1126/science.1151174

[10] Willems M, Genevieve D, Borck G, Baumann C, Baujat G, et al (2010) Molecular analysis of pericentrin gene (PCNT) in a series of 24 Seckel/microcephalic osteodysplastic primordial dwarfism type II (MOPD II) families. Journal of medical genetics 47: 797–802. 19643772 10.1136/jmg.2009.067298

[11] Hatch EM, Kulukian A, Holland AJ, Cleveland DW, Stearns T (2010) Cep152 interacts with Plk4 and is required for centriole duplication. The Journal of cell biology 191: 721–729. 21059850 10.1083/jcb.201006049 PMC2983069

[12] Cizmecioglu O, Arnold M, Bahtz R, Settele F, Ehret L, et al (2010) Cep152 acts as a scaffold for recruitment of Plk4 and CPAP to the centrosome. The Journal of cell biology 191: 731–739. 21059844 10.1083/jcb.201007107 PMC2983070

[13] Leal GF, Roberts E, Silva EO, Costa SM, Hampshire DJ, et al (2003) A novel locus for autosomal recessive primary microcephaly (MCPH6) maps to 13q12.2. J Med Genet 40: 540–542. 12843329 10.1136/jmg.40.7.540 PMC1735531

[14] Gul A, Hassan MJ, Hussain S, Raza SI, Chishti MS, et al (2006) A novel deletion mutation in CENPJ gene in a Pakistani family with autosomal recessive primary microcephaly. J Hum Genet 51: 760–764. 16900296 10.1007/s10038-006-0017-1

[15] Bond J, Roberts E, Springell K, Lizarraga SB, Scott S, et al (2005) A centrosomal mechanism involving CDK5RAP2 and CENPJ controls brain size. Nat Genet 37: 353–355. 15793586 10.1038/ng1539

[16] Kaindl AM, Passemard S, Kumar P, Kraemer N, Issa L, et al (2010) Many roads lead to primary autosomal recessive microcephaly. Progress in neurobiology 90: 363–383. 19931588 10.1016/j.pneurobio.2009.11.002

[17] Delaval B, Doxsey SJ (2010) Pericentrin in cellular function and disease. The Journal of cell biology 188: 181–190. 19951897 10.1083/jcb.200908114 PMC2812529

[18] Hung LY, Tang CJ, Tang TK (2000) Protein 4.1 R-135 interacts with a novel centrosomal protein (CPAP) which is associated with the gamma-tubulin complex. Mol Cell Biol 20: 7813–7825. 11003675 10.1128/mcb.20.20.7813-7825.2000 PMC86375

[19] Kohlmaier G, Loncarek J, Meng X, McEwen BF, Mogensen MM, et al (2009) Overly long centrioles and defective cell division upon excess of the SAS-4-related protein CPAP. Current biology: CB 19: 1012–1018. 19481460 10.1016/j.cub.2009.05.018 PMC2993638

[20] Schmidt TI, Kleylein-Sohn J, Westendorf J, Le Clech M, Lavoie SB, et al (2009) Control of centriole length by CPAP and CP110. Current biology: CB 19: 1005–1011. 19481458 10.1016/j.cub.2009.05.016

[21] Tang CJ, Fu RH, Wu KS, Hsu WB, Tang TK (2009) CPAP is a cell-cycle regulated protein that controls centriole length. Nat Cell Biol 11: 825–831. 19503075 10.1038/ncb1889

[22] Nigg EA, Raff JW (2009) Centrioles, centrosomes, and cilia in health and disease. Cell 139: 663–678. 19914163 10.1016/j.cell.2009.10.036

[23] Zyss D, Gergely F (2009) Centrosome function in cancer: guilty or innocent? Trends in cell biology 19: 334–346. 19570677 10.1016/j.tcb.2009.04.001

[24] Ganem NJ, Godinho SA, Pellman D (2009) A mechanism linking extra centrosomes to chromosomal instability. Nature 460: 278–282. 19506557 10.1038/nature08136 PMC2743290

[25] Murga M, Bunting S, Montana MF, Soria R, Mulero F, et al (2009) A mouse model of ATR-Seckel shows embryonic replicative stress and accelerated aging. Nature genetics 41: 891–898. 19620979 10.1038/ng.420 PMC2902278

[26] Skarnes WC, Rosen B, West AP, Koutsourakis M, Bushell W, et al (2011) A conditional knockout resource for the genome-wide study of mouse gene function. Nature 474: 337–342. 21677750 10.1038/nature10163 PMC3572410

[27] Sir JH, Barr AR, Nicholas AK, Carvalho OP, Khurshid M, et al (2011) A primary microcephaly protein complex forms a ring around parental centrioles. Nature genetics 43: 1147–1153. 21983783 10.1038/ng.971 PMC3299569

[28] Fitzgerald B, O'Driscoll M, Chong K, Keating S, Shannon P (2012) Neuropathology of fetal stage Seckel syndrome: a case report providing a morphological correlate for the emerging molecular mechanisms. Brain & development 34: 238–243. 21669506 10.1016/j.braindev.2011.05.007

[29] Hori A, Tamagawa K, Eber SW, Westmeier M, Hansmann I (1987) Neuropathology of Seckel syndrome in fetal stage with evidence of intrauterine developmental retardation. Acta neuropathologica 74: 397–401. 3687392 10.1007/BF00687219

[30] Arnold SR, Spicer D, Kouseff B, Lacson A, Gilbert-Barness E (1999) Seckel-like syndrome in three siblings. Pediatric and developmental pathology: the official journal of the Society for Pediatric Pathology and the Paediatric Pathology Society 2: 180–187. 10.1007/s100249900107 9949225

[31] Endoh-Yamagami S, Karkar KM, May SR, Cobos I, Thwin MT, et al (2010) A mutation in the pericentrin gene causes abnormal interneuron migration to the olfactory bulb in mice. Developmental biology 340: 41–53. 20096683 10.1016/j.ydbio.2010.01.017

[32] Carfagnini F, Tani G, Ambrosetto P (1999) MR findings in Seckel's syndrome: report of a case. Pediatric radiology 29: 849–850. 10552067 10.1007/s002470050711

[33] Abuelo D (2007) Microcephaly syndromes. Seminars in pediatric neurology 14: 118–127. 17980308 10.1016/j.spen.2007.07.003

[34] Capovilla G, Lorenzetti ME, Montagnini A, Borgatti R, Piccinelli P, et al (2001) Seckel's syndrome and malformations of cortical development: report of three new cases and review of the literature. Journal of child neurology 16: 382–386. 11392528 10.1177/088307380101600516

[35] Shanske A, Caride DG, Menasse-Palmer L, Bogdanow A, Marion RW (1997) Central nervous system anomalies in Seckel syndrome: report of a new family and review of the literature. American journal of medical genetics 70: 155–158. 9128935 10.1002/(sici)1096-8628(19970516)70:2<155::aid-ajmg10>3.0.co;2-i

[36] Gruber R, Zhou Z, Sukchev M, Joerss T, Frappart PO, et al (2011) MCPH1 regulates the neuroprogenitor division mode by coupling the centrosomal cycle with mitotic entry through the Chk1-Cdc25 pathway. Nature cell biology 13: 1325–1334. 21947081 10.1038/ncb2342

[37] Lizarraga SB, Margossian SP, Harris MH, Campagna DR, Han AP, et al (2010) Cdk5rap2 regulates centrosome function and chromosome segregation in neuronal progenitors. Development 137: 1907–1917. 20460369 10.1242/dev.040410 PMC2867323

[38] Sanchez-Andrade G, Kendrick KM (2011) Roles of alpha- and beta-estrogen receptors in mouse social recognition memory: effects of gender and the estrous cycle. Hormones and behavior 59: 114–122. 21056567 10.1016/j.yhbeh.2010.10.016

[39] Kogan JH, Frankland PW, Silva AJ (2000) Long-term memory underlying hippocampus-dependent social recognition in mice. Hippocampus 10: 47–56. 10706216 10.1002/(SICI)1098-1063(2000)10:1<47::AID-HIPO5>3.0.CO;2-6

[40] Engelmann M, Hadicke J, Noack J (2011) Testing declarative memory in laboratory rats and mice using the nonconditioned social discrimination procedure. Nature protocols 6: 1152–1162. 21799485 10.1038/nprot.2011.353

[41] Griffith E, Walker S, Martin CA, Vagnarelli P, Stiff T, et al (2008) Mutations in pericentrin cause Seckel syndrome with defective ATR-dependent DNA damage signaling. Nature genetics 40: 232–236. 18157127 10.1038/ng.2007.80 PMC2397541

[42] Polo SE, Jackson SP (2011) Dynamics of DNA damage response proteins at DNA breaks: a focus on protein modifications. Genes & development 25: 409–433. 21363960 10.1101/gad.2021311 PMC3049283

[43] Zhivotovsky B, Kroemer G (2004) Apoptosis and genomic instability. Nature reviews Molecular cell biology 5: 752–762. 15340382 10.1038/nrm1443

[44] Adiyaman P, Berberoglu M, Aycan Z, Evliyaoglu O, Ocal G (2004) Seckel-like syndrome: a patient with precocious puberty associated with nonclassical congenital adrenal hyperplasia. Journal of pediatric endocrinology & metabolism: JPEM 17: 105–110. 14960029 10.1515/jpem.2004.17.1.105

[45] Stoppoloni G, Stabile M, Rinaldi MM, Prisco F, Rabuano RG, et al (1992) Seckel syndrome: report of three sibships with the type I primordial dwarfism. Possible linkage with HLA locus. Annales de genetique 35: 213–216. 1296517

[46] Daughaday W (1941) A comparison of the X-zone of the adrenal cortex in two inbred strains of mice. Cancer Research 1: 883–885.

[47] Reddy S, Starr C (2007) Seckel syndrome and spontaneously dislocated lenses. Journal of cataract and refractive surgery 33: 910–912. 17466870 10.1016/j.jcrs.2006.12.027

[48] Guirgis MF, Lam BL, Howard CW (2001) Ocular manifestations of Seckel syndrome. American journal of ophthalmology 132: 596–597. 11589896 10.1016/s0002-9394(01)01046-7

[49] Can E, Bulbul A, Uslu S, Demirin H, Comert S, et al (2010) A case of Seckel syndrome with Tetralogy of Fallot. Genetic counseling 21: 49–51. 20420029

[50] Ucar B, Kilic Z, Dinleyici EC, Yakut A, Dogruel N (2004) Seckel syndrome associated with atrioventricular canal defect: a case report. Clinical dysmorphology 13: 53–55. 15127771 10.1097/00019605-200401000-00017

[51] Rappen U, von Brenndorff AI (1993) [Cardiac symptoms in 2 patients with Seckel syndrome]. Monatsschrift Kinderheilkunde: Organ der Deutschen Gesellschaft fur Kinderheilkunde 141: 584–586. 8413337

[52] Howanietz H, Frisch H, Jedlicka-Kohler I, Steger H (1989) [Seckel dwarfism based on a personal case]. Klinische Padiatrie 201: 139–141. 2716236 10.1055/s-2007-1025292

[53] Fukuda S, Morishita Y, Hashiguchi M, Taira A (1991) [Seckel's syndrome associated with atrial septal defect: a case report and review of the literature in Japan]. Kyobu geka The Japanese journal of thoracic surgery 44: 411–413. 2051684

[54] Elwell M, Mahler J (1999) Pathology of the Mouse; Maronpot R, editor: Cache River Press.

[55] Liu Z, Yue S, Chen X, Kubin T, Braun T (2010) Regulation of cardiomyocyte polyploidy and multinucleation by CyclinG1. Circulation research 106: 1498–1506. 20360255 10.1161/CIRCRESAHA.109.211888

[56] Keenan CM, Vidal JD (2006) Standard morphologic evaluation of the heart in the laboratory dog and monkey. Toxicologic pathology 34: 67–74. 16507546 10.1080/01926230500369915

[57] Cho JH, Chang CJ, Chen CY, Tang TK (2006) Depletion of CPAP by RNAi disrupts centrosome integrity and induces multipolar spindles. Biochem Biophys Res Commun 339: 742–747. 16316625 10.1016/j.bbrc.2005.11.074

[58] Mayer TU, Kapoor TM, Haggarty SJ, King RW, Schreiber SL, et al (1999) Small molecule inhibitor of mitotic spindle bipolarity identified in a phenotype-based screen. Science 286: 971–974. 10542155 10.1126/science.286.5441.971

[59] Gergely F, Basto R (2008) Multiple centrosomes: together they stand, divided they fall. Genes & development 22: 2291–2296. 18765784 10.1101/gad.1715208 PMC2749672

[60] Alderton GK, Joenje H, Varon R, Borglum AD, Jeggo PA, et al (2004) Seckel syndrome exhibits cellular features demonstrating defects in the ATR-signalling pathway. Human molecular genetics 13: 3127–3138. 15496423 10.1093/hmg/ddh335

[61] Cimprich KA, Cortez D (2008) ATR: an essential regulator of genome integrity. Nature reviews Molecular cell biology 9: 616–627. 18594563 10.1038/nrm2450 PMC2663384

[62] Pommier Y (2006) Topoisomerase I inhibitors: camptothecins and beyond. Nature reviews Cancer 6: 789–802. 16990856 10.1038/nrc1977

[63] O'Driscoll M, Gennery AR, Seidel J, Concannon P, Jeggo PA (2004) An overview of three new disorders associated with genetic instability: LIG4 syndrome, RS-SCID and ATR-Seckel syndrome. DNA repair 3: 1227–1235. 15279811 10.1016/j.dnarep.2004.03.025

[64] Kjaer I, Hansen N, Becktor KB, Birkebaek N, Balslev T (2001) Craniofacial morphology, dentition, and skeletal maturity in four siblings with Seckel syndrome. The Cleft palate-craniofacial journal: official publication of the American Cleft Palate-Craniofacial Association 38: 645–651. 11681999 10.1597/1545-1569_2001_038_0645_cmdasm_2.0.co_2

[65] Gotz M, Huttner WB (2005) The cell biology of neurogenesis. Nature reviews Molecular cell biology 6: 777–788. 16314867 10.1038/nrm1739

[66] Lu B, Jan L, Jan YN (2000) Control of cell divisions in the nervous system: symmetry and asymmetry. Annual review of neuroscience 23: 531–556. 10.1146/annurev.neuro.23.1.531 10845074

[67] Evans PD, Vallender EJ, Lahn BT (2006) Molecular evolution of the brain size regulator genes CDK5RAP2 and CENPJ. Gene 375: 75–79. 16631324 10.1016/j.gene.2006.02.019

[68] Crasta K, Ganem NJ, Dagher R, Lantermann AB, Ivanova EV, et al (2012) DNA breaks and chromosome pulverization from errors in mitosis. Nature 482: 53–58. 22258507 10.1038/nature10802 PMC3271137

[69] Rogakou EP, Nieves-Neira W, Boon C, Pommier Y, Bonner WM (2000) Initiation of DNA fragmentation during apoptosis induces phosphorylation of H2AX histone at serine 139. The Journal of biological chemistry 275: 9390–9395. 10734083 10.1074/jbc.275.13.9390

[70] Basto R, Lau J, Vinogradova T, Gardiol A, Woods CG, et al (2006) Flies without centrioles. Cell 125: 1375–1386. 16814722 10.1016/j.cell.2006.05.025

[71] Dobbelaere J, Josue F, Suijkerbuijk S, Baum B, Tapon N, et al (2008) A genome-wide RNAi screen to dissect centriole duplication and centrosome maturation in Drosophila. PLoS biology 6: e224. 18798690 10.1371/journal.pbio.0060224 PMC2535660

[72] Graser S, Stierhof YD, Lavoie SB, Gassner OS, Lamla S, et al (2007) Cep164, a novel centriole appendage protein required for primary cilium formation. The Journal of cell biology 179: 321–330. 17954613 10.1083/jcb.200707181 PMC2064767

[73] Uetake Y, Sluder G (2010) Prolonged prometaphase blocks daughter cell proliferation despite normal completion of mitosis. Current biology: CB 20: 1666–1671. 20832310 10.1016/j.cub.2010.08.018 PMC2946429

[74] Orth JD, Loewer A, Lahav G, Mitchison TJ (2012) Prolonged mitotic arrest triggers partial activation of apoptosis, resulting in DNA damage and p53 induction. Molecular biology of the cell 23: 567–576. 22171325 10.1091/mbc.E11-09-0781 PMC3279386

[75] Janssen A, van der Burg M, Szuhai K, Kops GJ, Medema RH (2011) Chromosome segregation errors as a cause of DNA damage and structural chromosome aberrations. Science 333: 1895–1898. 21960636 10.1126/science.1210214

[76] Storchova Z, Pellman D (2004) From polyploidy to aneuploidy, genome instability and cancer. Nature reviews Molecular cell biology 5: 45–54. 14708009 10.1038/nrm1276

[77] Borel F, Lohez OD, Lacroix FB, Margolis RL (2002) Multiple centrosomes arise from tetraploidy checkpoint failure and mitotic centrosome clusters in p53 and RB pocket protein-compromised cells. Proceedings of the National Academy of Sciences of the United States of America 99: 9819–9824. 12119403 10.1073/pnas.152205299 PMC125028

[78] Thoolen B, Maronpot RR, Harada T, Nyska A, Rousseaux C, et al (2010) Proliferative and nonproliferative lesions of the rat and mouse hepatobiliary system. Toxicologic pathology 38: 5S–81S. 21191096 10.1177/0192623310386499

[79] Hoeijmakers JH (2009) DNA damage, aging, and cancer. The New England journal of medicine 361: 1475–1485. 19812404 10.1056/NEJMra0804615

[80] Jeffery AN, Metcalf BS, Hosking J, Streeter AJ, Voss LD, et al (2012) Age before stage: insulin resistance rises before the onset of puberty: a 9-year longitudinal study (EarlyBird 26). Diabetes care 35: 536–541. 22279034 10.2337/dc11-1281 PMC3322712

[81] Huang-Doran I, Bicknell LS, Finucane FM, Rocha N, Porter KM, et al (2011) Genetic defects in human pericentrin are associated with severe insulin resistance and diabetes. Diabetes 60: 925–935. 21270239 10.2337/db10-1334 PMC3046854

[82] Klingseisen A, Jackson AP (2011) Mechanisms and pathways of growth failure in primordial dwarfism. Genes & development 25: 2011–2024. 21979914 10.1101/gad.169037 PMC3197200

[83] Jurczyk A, Pino SC, O'Sullivan-Murphy B, Addorio M, Lidstone EA, et al (2010) A novel role for the centrosomal protein, pericentrin, in regulation of insulin secretory vesicle docking in mouse pancreatic beta-cells. PloS one 5: e11812. 20676397 10.1371/journal.pone.0011812 PMC2910730

[84] Richtsmeier JT, Baxter LL, Reeves RH (2000) Parallels of craniofacial maldevelopment in Down syndrome and Ts65Dn mice. Developmental dynamics: an official publication of the American Association of Anatomists 217: 137–145. 10706138 10.1002/(SICI)1097-0177(200002)217:2<137::AID-DVDY1>3.0.CO;2-N

[85] Mahajan VB, Skeie JM, Assefnia AH, Mahajan M, Tsang SH (2011) Mouse eye enucleation for remote high-throughput phenotyping. Journal of visualized experiments: JoVE 57: e3184. 10.3791/3184 PMC3308585 22126835

[86] Hancock JM, Adams NC, Aidinis V, Blake A, Bogue M, et al (2007) Mouse Phenotype Database Integration Consortium: integration [corrected] of mouse phenome data resources. Mammalian genome: official journal of the International Mammalian Genome Society 18: 157–163. 17436037 10.1007/s00335-007-9004-x PMC4230762

[87] Masuya H, Inoue M, Wada Y, Shimizu A, Nagano J, et al (2005) Implementation of the modified-SHIRPA protocol for screening of dominant phenotypes in a large-scale ENU mutagenesis program. Mammalian genome: official journal of the International Mammalian Genome Society 16: 829–837. 16284798 10.1007/s00335-005-2430-8

[88] Dertinger SD, Torous DK, Tometsko KR (1996) Simple and reliable enumeration of micronucleated reticulocytes with a single-laser flow cytometer. Mutation research 371: 283–292. 9008730 10.1016/s0165-1218(96)90117-2

[89] Pierce MJ, Morse RP (2012) The neurologic findings in Taybi-Linder syndrome (MOPD I/III): case report and review of the literature. American journal of medical genetics Part A 158A: 606–610. 22302400 10.1002/ajmg.a.33958

[90] Loizou JI, Sancho R, Kanu N, Bolland DJ, Yang F, et al (2011) ATMIN is required for maintenance of genomic stability and suppression of B cell lymphoma. Cancer Cell 19: 587–600. 21575860 10.1016/j.ccr.2011.03.022 PMC4452547

[91] Shannon P, Markiel A, Ozier O, Baliga NS, Wang JT, et al (2003) Cytoscape: a software environment for integrated models of biomolecular interaction networks. Genome research 13: 2498–2504. 14597658 10.1101/gr.1239303 PMC403769

[92] Martin A, Ochagavia ME, Rabasa LC, Miranda J, Fernandez-de-Cossio J, et al (2010) BisoGenet: a new tool for gene network building, visualization and analysis. BMC bioinformatics 11: 91. 20163717 10.1186/1471-2105-11-91 PMC3098113

[93] Assenov Y, Ramirez F, Schelhorn SE, Lengauer T, Albrecht M (2008) Computing topological parameters of biological networks. Bioinformatics 24: 282–284. 18006545 10.1093/bioinformatics/btm554

[94] Kamburov A, Wierling C, Lehrach H, Herwig R (2009) ConsensusPathDB–a database for integrating human functional interaction networks. Nucleic acids research 37: D623–628. 18940869 10.1093/nar/gkn698 PMC2686562

[95] Team RDC (2011) R: A language and environment for statistical computing. Vienna, Austria.: R Foundation for Statistical Computing.

