# Supplementary material for: Disruption of Mouse Cenpj, a Regulator of Centriole Biogenesis, Phenocopies Seckel Syndrome
Source: PLoS Genet. 2012 Nov 15;8(11):e1003022. doi: 10.1371/journal.pgen.1003022 (PMC3499256; doi:10.1371/journal.pgen.1003022)
Supplement: Figure S1 — A. Design and validation of the Cenpj allele. The L1L2_gt1 cassette was inserted at basepair 57174548 of chromosome 14 upstream of a Cenpj critical exon (exon 5, Build 37). The cassette is composed of an FRT-flanked lacZ/neomycin sequence followed by a loxP site. An additional loxP site is inserted downstream of the targeted exon at basepair 57173663. The critical exon is thus flanked by loxP sites. Further information on targeting strategies used for this and other KOMP alleles can be found at http://www.knockoutmouse.org/aboutkompstrategies. B. Correct targeting in founder mice was confirmed by standard PCR using the primers shown in Figure S1D (for more details on cassette quality control see http://www.knockoutmouse.org/kb/entry/90/). Gels show the presence of LacZ, 5′FRT and LoxP sites, generation of a mutant band (MUT) and absence of backbone (VF4). C. Correct targeting was also confirmed by loss of wildtype allele qPCR. A TaqMan qPCR assay was designed to the wildtype sequence removed during recombineering of the mutant allele. Samples were amplified in a multiplex reaction with a Tfrc endogenous VIC labeled control (Applied Biosystems) and then compared to known wildtype controls using the ΔΔCt method. Loss of one copy in heterozygotes and no amplification at all in homozygotes strongly suggests that the targeting is correct. No loss in copy number would indicate either a wildtype mouse (confirmed by neo count qPCR) or an incorrect targeting event. Targeting was also confirmed by traditional end point PCR by a failure in homozygotes (detected by neo count qPCR) to amplify a product designed to the wild-type allele, using primers flanking the cassette insertion point. D. Primers used for quality control and genotyping. (PDF) [file pgen.1003022.s001.pdf]

McIntyre et al., Supp Fig. 1

A

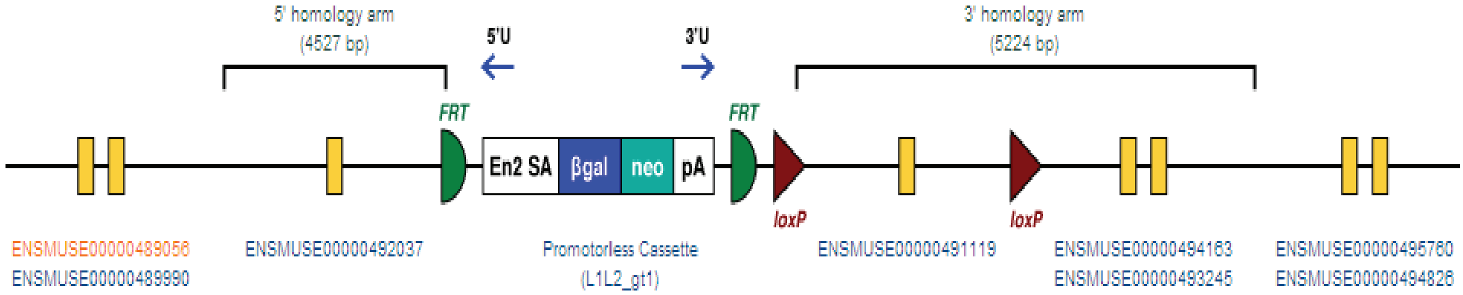

B

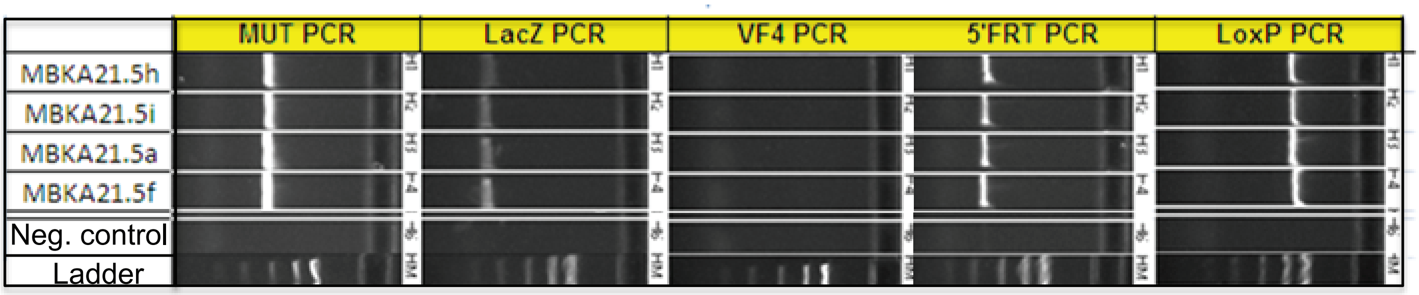

C

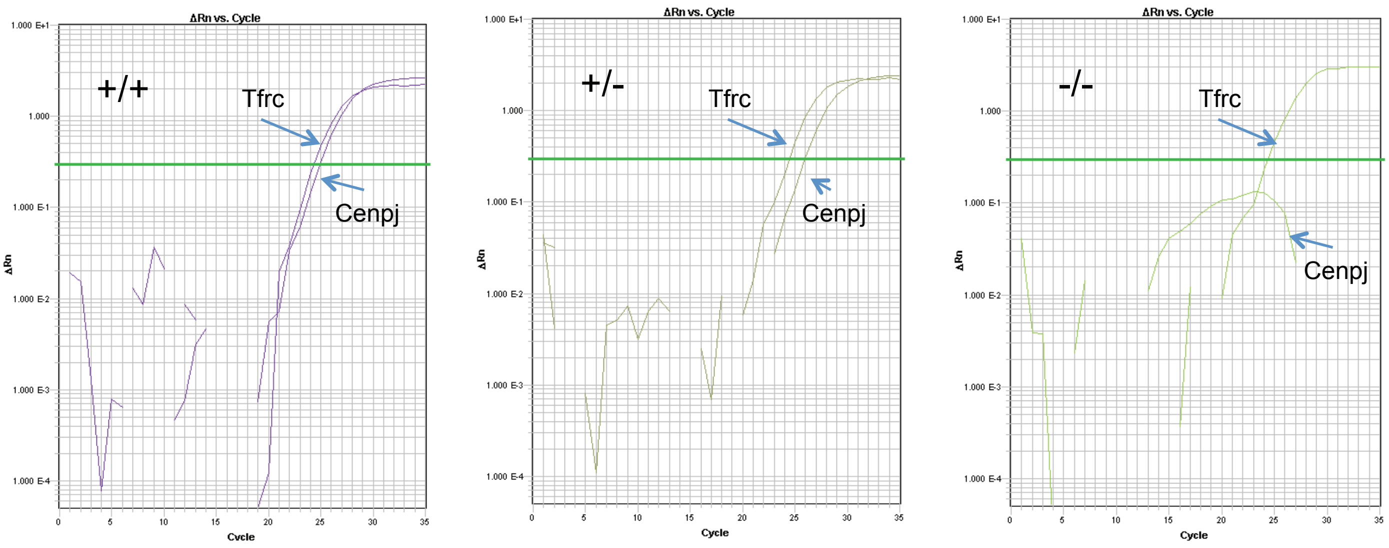

MBKA60.3c WT (used as calibrator).
Cenpj and Tfrc endogenous control Ct ~ 1 cycle apart

MBKA69.1c heterozygote.
Cenpj and Tfrc endogenous control Ct ~ 2 cycles apart

MBKA69.1e homozygote.
No amplification of Cenpj assay above the Ct detection threshold

D

PCR	F PRIMER	R PRIMER
**Vector Backbone (VF4) PCR**	GTAGCTGACATTCATCCGGG	CTTTTCTACGGGGTCTGACG
**Cenpj WT PCR**	TCAAGCTATTTTGGCTCCACAG	TTGATGATTCCCAGCACCAC
**Cenpj Mut PCR**	TCAAGCTATTTTGGCTCCACAG	TCGTGGTATCGTTATGCGCC
**LacZ PCR**	ATCACGACGCGCTGTATC	ACATCGGGCAAATAATATCG
**5' FRT PCR**	AGGCGCATAACGATACCACGAT	CCACAACGGGTTCTTCTGTT
**LoxP PCR**	ATCCGGGGGTACCGCGTCGAG	ACTGATGGCGAGCTCAGACC
**WT LoA qPCR**	GCAGAAGGTGTCCAGTACAATGT	GCAAGAGACTGTGGTTGGAAGTAAG
