# Supplementary material for: Disruption of Mouse Cenpj, a Regulator of Centriole Biogenesis, Phenocopies Seckel Syndrome
Source: PLoS Genet. 2012 Nov 15;8(11):e1003022. doi: 10.1371/journal.pgen.1003022 (PMC3499256; doi:10.1371/journal.pgen.1003022)

A

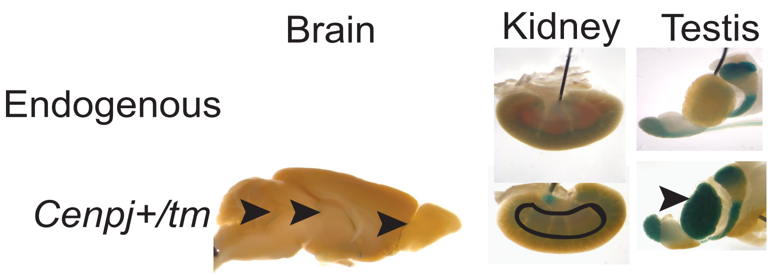

B

Exons spliced	Relative to wild-type (%)		Relative to wild-type exons one and two (%)	
	WT	*tm/tm*	WT	*tm/tm*
3 to 6	100±17	444±95	0.35±0.2	1.5±0.3
4 to 5	100±19	2.1±0.5	144±31	2.9±0.7
4 to 6	100±26	2.1±0.5	40±9	0.9±0.2

C

Truncated Cenpj (spliced Ex3-6)	MFLMPTSSELNSGQNFLTQWMTSPSRAGVILNRGFPILEADDKQA<br>ATNVSTSFPAKATHFSNSFSISSEEDSFHEEQKLEAGGPYKPWSE<br>NPEAPPVFPSVRKEPIASRQDAPGCQEDNNNDLTPHLESEFKEVA<br>NKNPLFKKLEQLKEIQQKKQEQLKRQQLEQLQRLMEEQEKLLTMV<br>SAQHAFPGPSKLLLERSSRHLRTT**Stop**KSRSNWKSGSLDRSSC
Truncated Cenpj (spliced Ex4-6)	MFLMPTSSELNSGQNFLTQWMTSPSRAGVILNRGFPILEADDKQA<br>ATNVSTSFPAKATHFSNSFSISSEEDSFHEEQKLEAGGPYKPWSE<br>NPEAPPVFPSVRKEPIASRQDAPGCQEDNNNDLTPHLESEFKEVA<br>NKNPLFKKLEQLKEIQQKKQEQLKRQQLEQLQRLMEEQEKLLTMV<br>SAQHAFPGTLLPDDQSQKHRSPGDLTLPPHSYSNPTQENSCASNV<br>LPDEQSNFCRATQDSVLTSKNASDLFYESQYQEAHVKRNDLKEES<br>PAHPSGPSKLLLERSSRHLRTT**Stop**KSRSNWKSGSLDRSSC

D

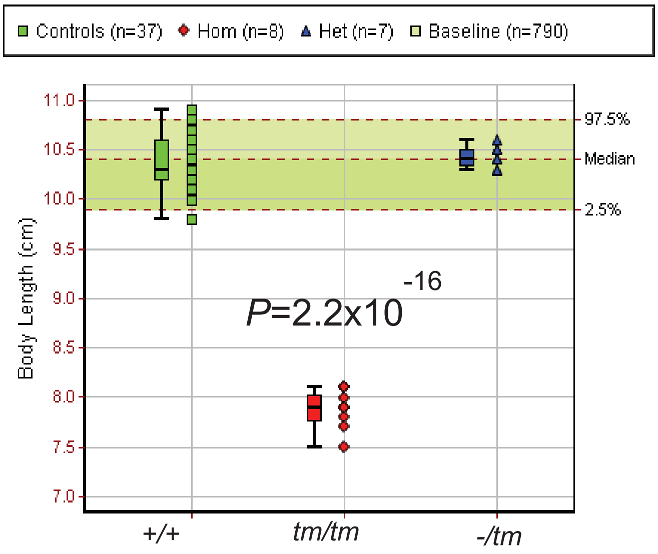

F

Karyotype	% of metaphases (n=20)	
	Cenpj+/+	*Cenpj*
2N	80	25
2N+numerical	10	10
2N+structural	5	0
4N	5	40
4N+numerical	0	15
4N+structural	0	5
>8N	0	5

G

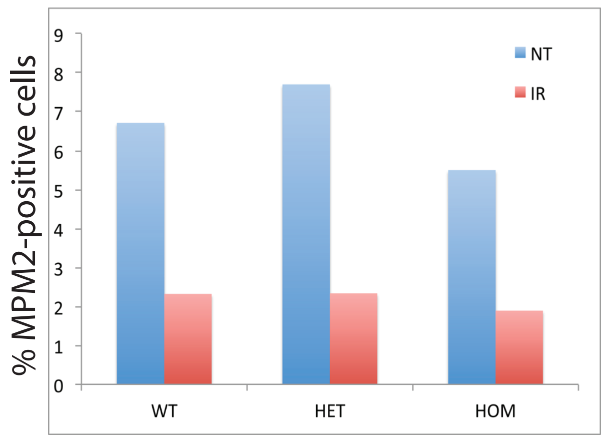

E

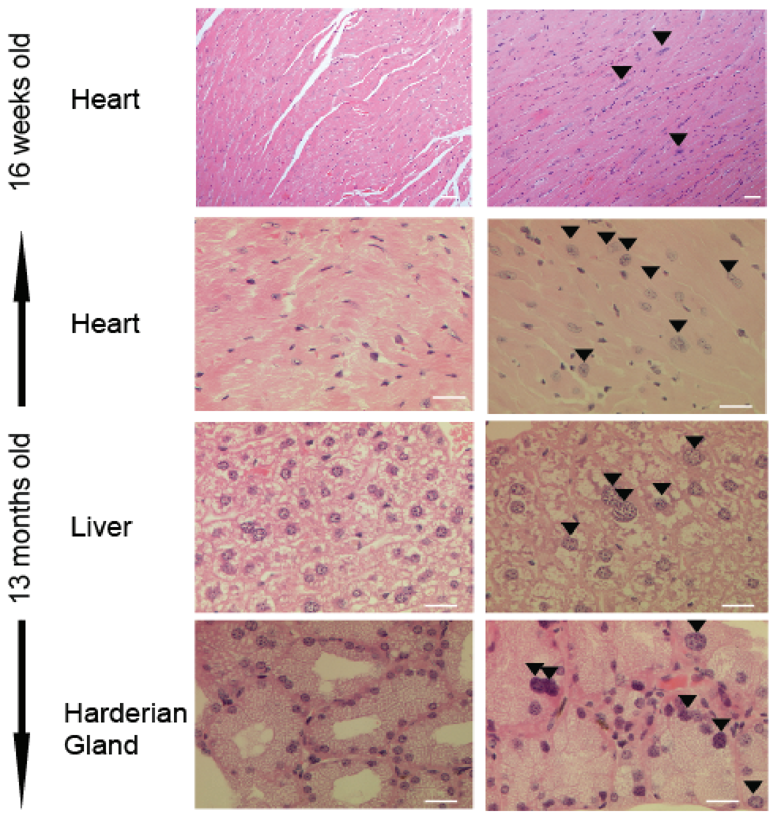

Supplement: Figure S2 — Analyses of Cenpjtm/tm mice A. LacZ staining in the brain was restricted to the lining of the cerebellar aqueduct and the fourth ventricle, which connected to a focus of staining at the precommissural nucleus. Weak staining was observed around the ventromedial preoptic nucleus. Strong LacZ staining was observed in the testes (the epididymus contains endogenous staining) and moderate staining in the medulla of the kidneys (the cortex contains background staining). B. Cryptic splicing of Cenpj. Percentages show Mean±S.E.M. expression of Cenpj across exon boundaries as determined by quantitative RT-PCR relative to Gapdh for Cenpjtm/tm relative to Cenpj+/+ for RNA extracted from n = 3 MEF lines. C. Cenpj coding begins in Exon 2. Blue font indicates alternate exons. Red font indicates amino acids encoded across a splice junction. Alternatively spliced transcripts (Ex3–6 and Ex4–6) result in truncated protein products. Yellow highlight indicates the point at which the protein goes out-of-frame resulting in a stop codon. D. Measurements of nose-to-tail base length of male Cenpj+/+ (n = 37), Cenpj+/tm (n = 7), Cenpjtm/tm (n = 8) and baseline wild-type control (n = 790) mice at 14 weeks of age. Data show that male Cenpjtm/tm mice are significantly shorter than Cenpj+/+ mice (*P = 2.2×10^−16^, t-test). The lower whisker extends to the lowest datum still within 1.5 Inter-quartile range (IQR) of the lower quartile. The upper whisker extends to the highest datum still within 1.5 IQR of the upper quartile. E. Representative haematoxylin and eosin stained sections. Karyomegaly (arrow heads) of cardiomyocytes was increased at 16 weeks of age in 5/6 Cenpjtm/tm and 1/6 Cenpj+/tm mice when compared to wild-type control (0/4; Cenpjtm/tm vs Cenpj+/+, P = 0.048, Fisher exact test). Histopathology at 13 months of age revealed an increased prevalence of karyomegaly in cardiomyocytes (17.6% (16/91) vs 3.3% (4/120)), hepatocytes (9.3% (10/108) vs 3.0% (3/101)) and cells of the Harderian glands [file pgen.1003022.s002.pdf]
