# Supplementary material for: Disruption of Mouse Cenpj, a Regulator of Centriole Biogenesis, Phenocopies Seckel Syndrome
Source: PLoS Genet. 2012 Nov 15;8(11):e1003022. doi: 10.1371/journal.pgen.1003022 (PMC3499256; doi:10.1371/journal.pgen.1003022)
Supplement: Figure S3 — Brain measurement analysis. Diagram to show the measurements of adult brains taken at 16 weeks of age. Thalamus, mammillothalamic tracts and caudate were used as markers. (PDF) [file pgen.1003022.s003.pdf]

McIntyre et al.,
Supplementary Fig. 3

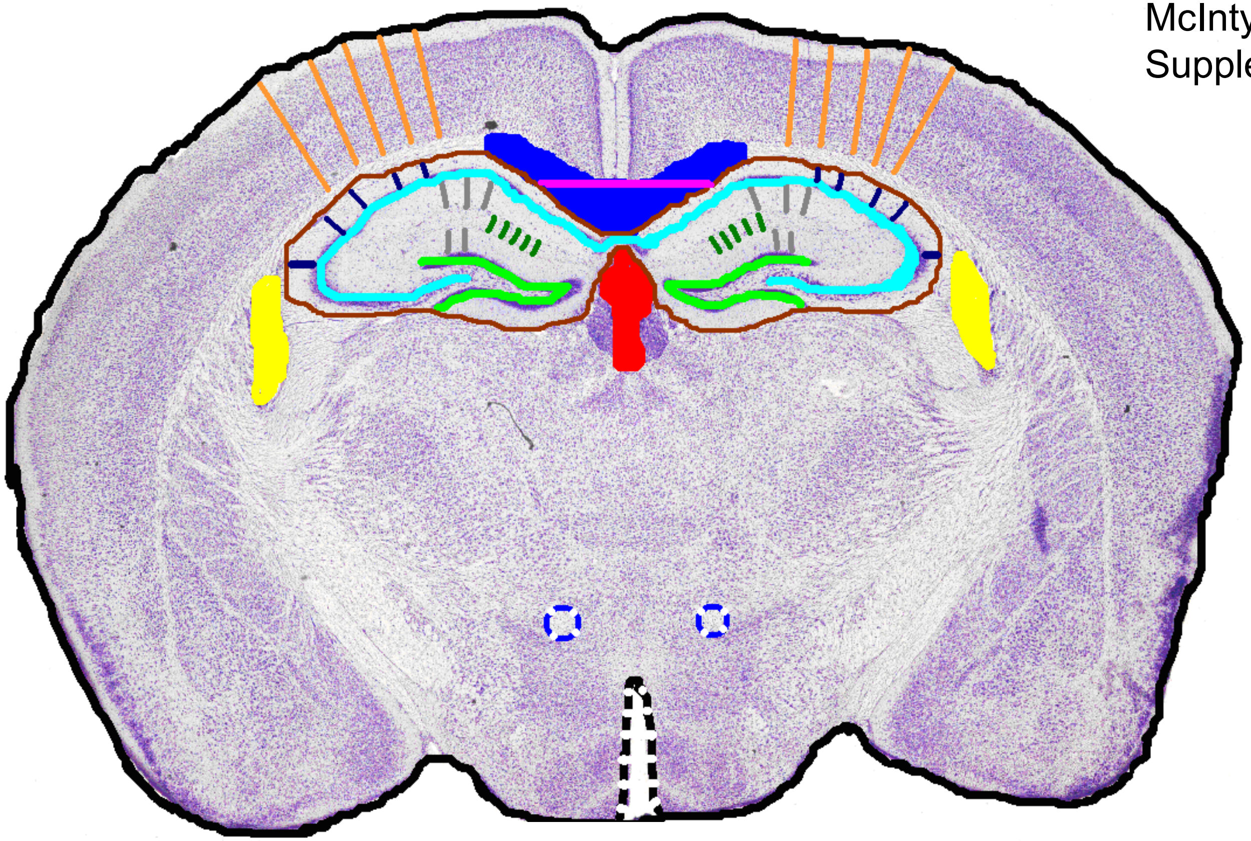

Dorsal 3rd ventricle area

Total lateral ventricle area

Corpus callosum area

Corpus callosum max span

Average cortical thickness

Thalamus

Total internal length of pyramidal cell layer of hippocampus

Average thickness of molecular layer of hippocampus

Average thickness of stratum radiatum of hippocampus

Average thickness of oriens layer of hippocampus

Total brain section area

Mammillothalamic tract

Caudate

Total internal length dentate gyrus
