# Supplementary figures and images for: Disruption of Mouse Cenpj, a Regulator of Centriole Biogenesis, Phenocopies Seckel Syndrome
Source: PLoS Genet. 2012 Nov 15;8(11):e1003022. doi: 10.1371/journal.pgen.1003022 (PMC3499256; doi:10.1371/journal.pgen.1003022)

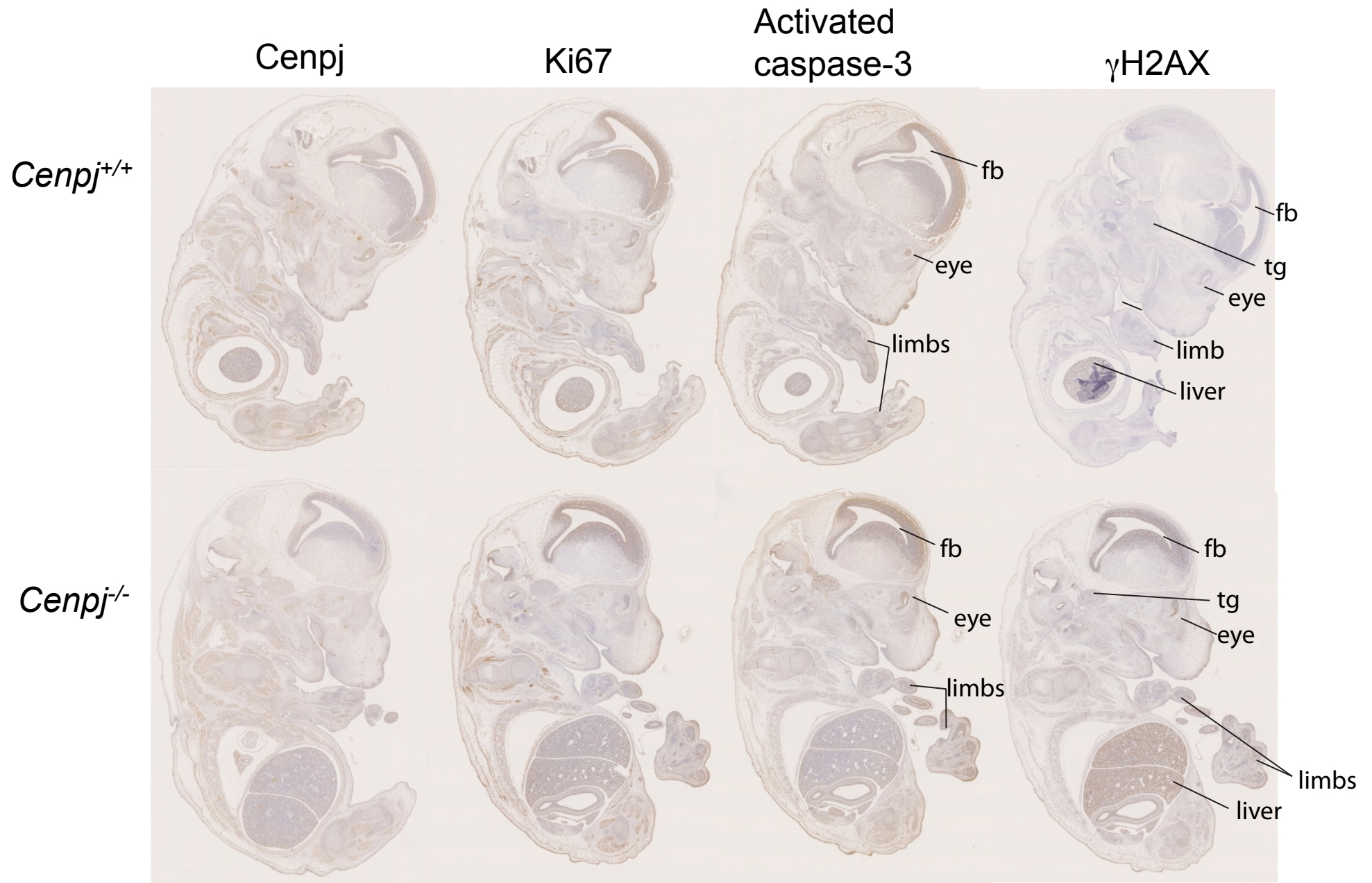

McIntyre et al., Supp. Fig 4.

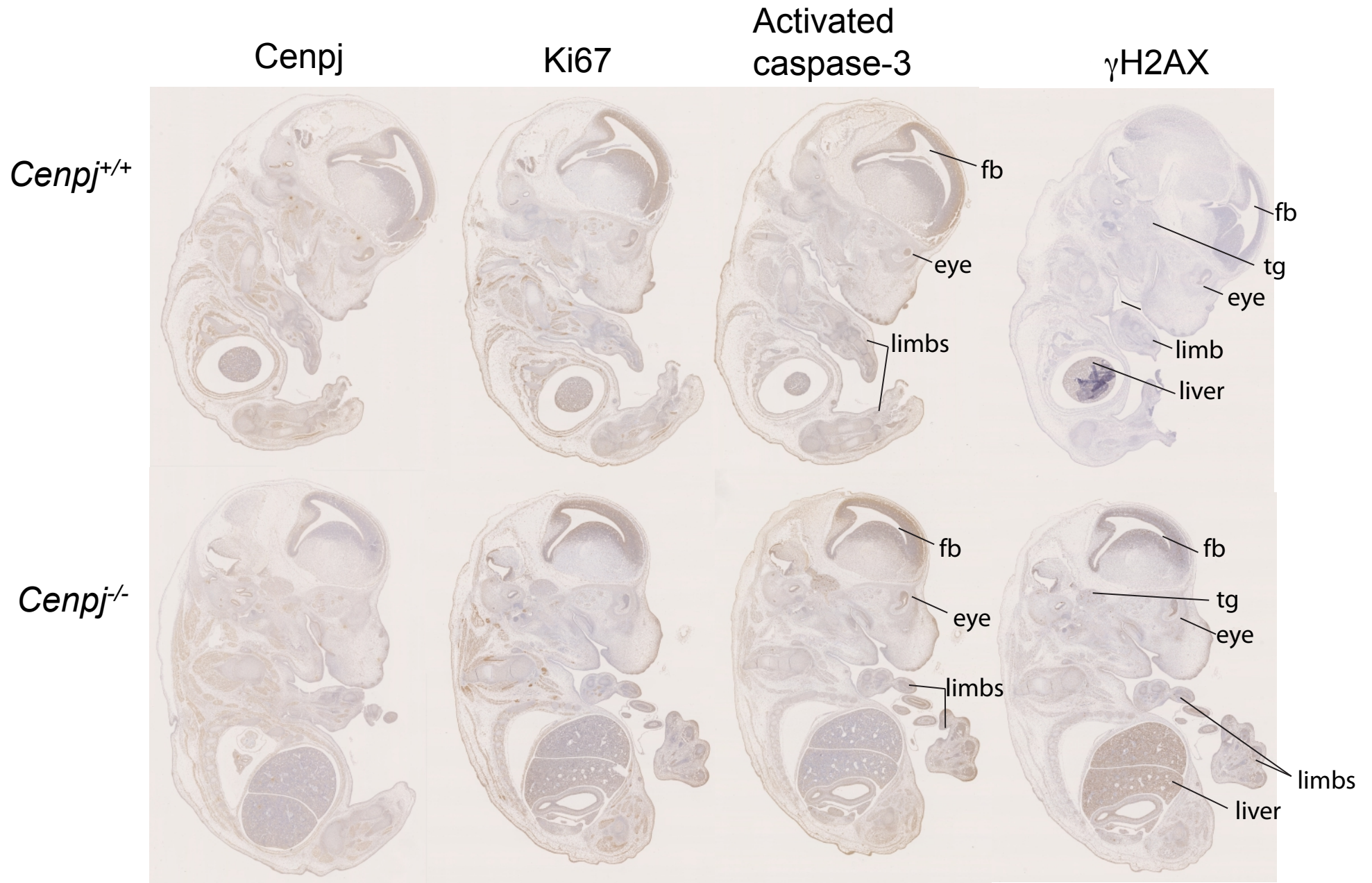

McIntyre et al., Supp. Fig 4.

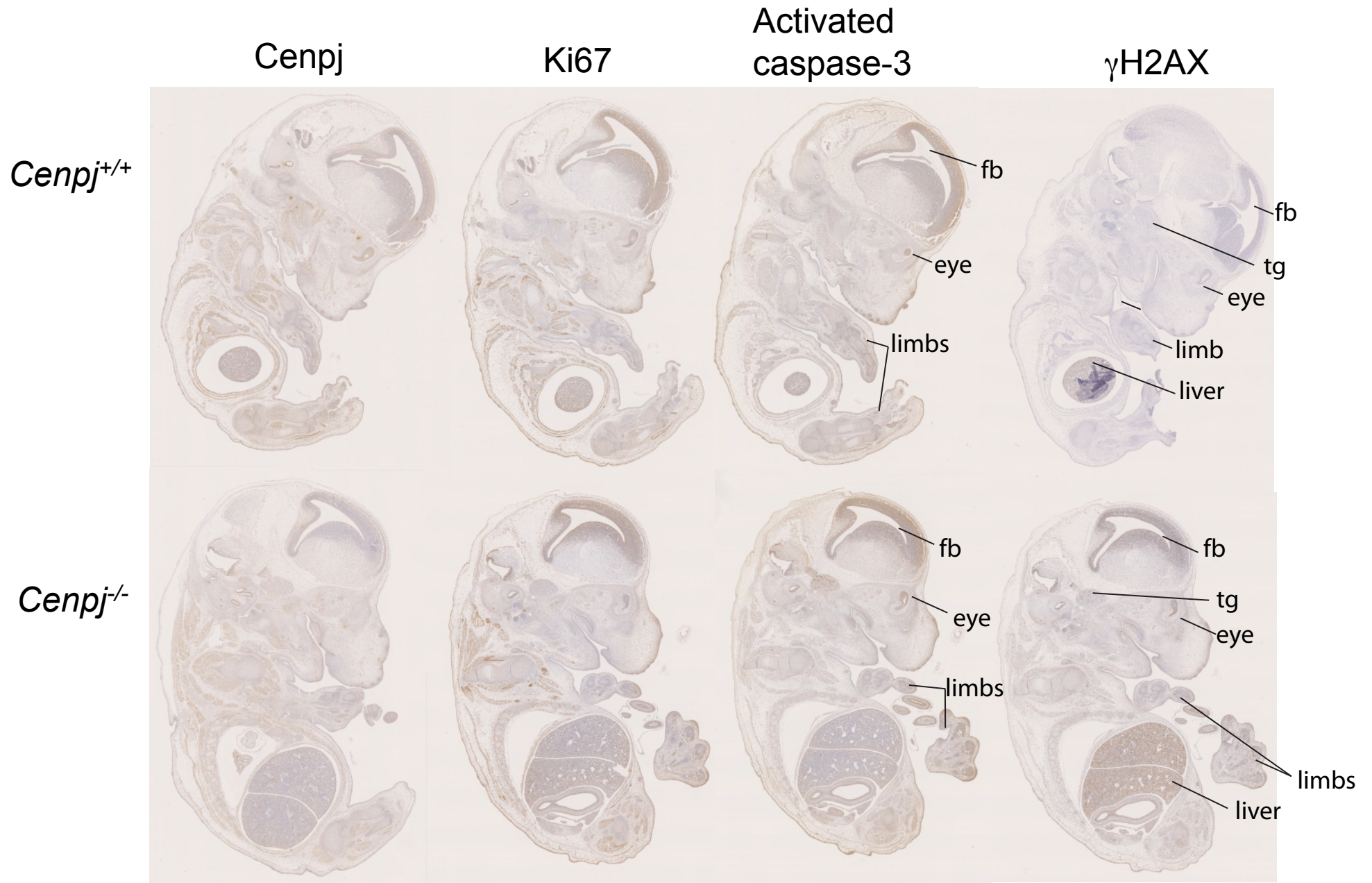

McIntyre et al., Supp. Fig 4.

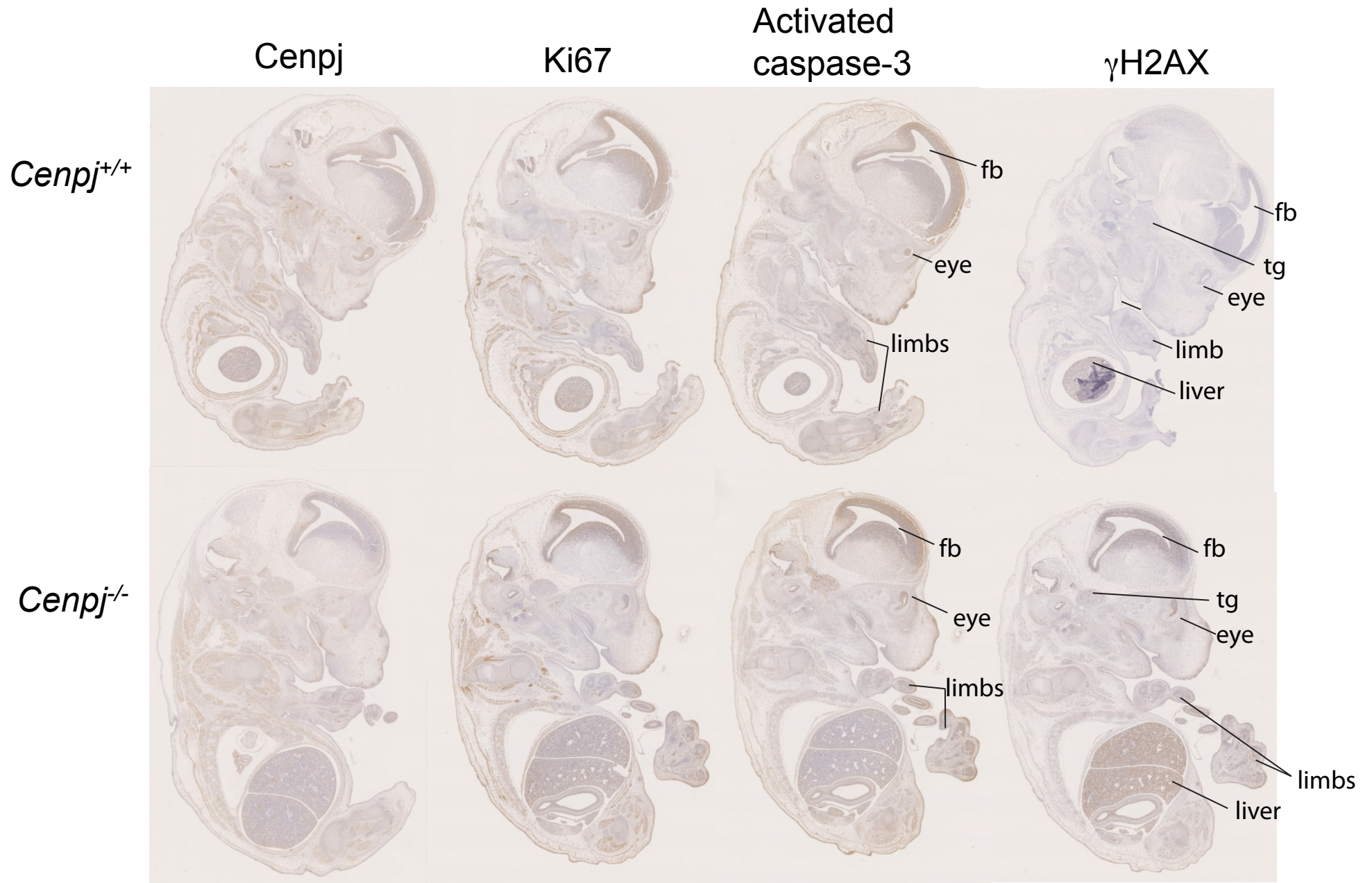

Supplement: Figure S4 — Apoptotic cells are scattered throughout Cenpjtm/tm embryos. Representative whole embryo (14.5 d.p.c.) images show immunohistochemical staining for Cenpj, Ki67 as a marker of proliferation, cleaved (activated) caspase-3 as a marker of apoptosis and Ser139-phosphorylated H2AX (γH2AX) as a marker of DNA damage. Apoptotic cells were scattered throughout embryos and this was more apparent in the forebrain (fb), eyes and limbs. The pattern of γH2AX-positive staining was similar to cleaved caspase-3 however this was also more apparent in the trigeminal ganglion (tg) and liver. Scale bar 500 µm. (PDF) [file pgen.1003022.s004.pdf]

A

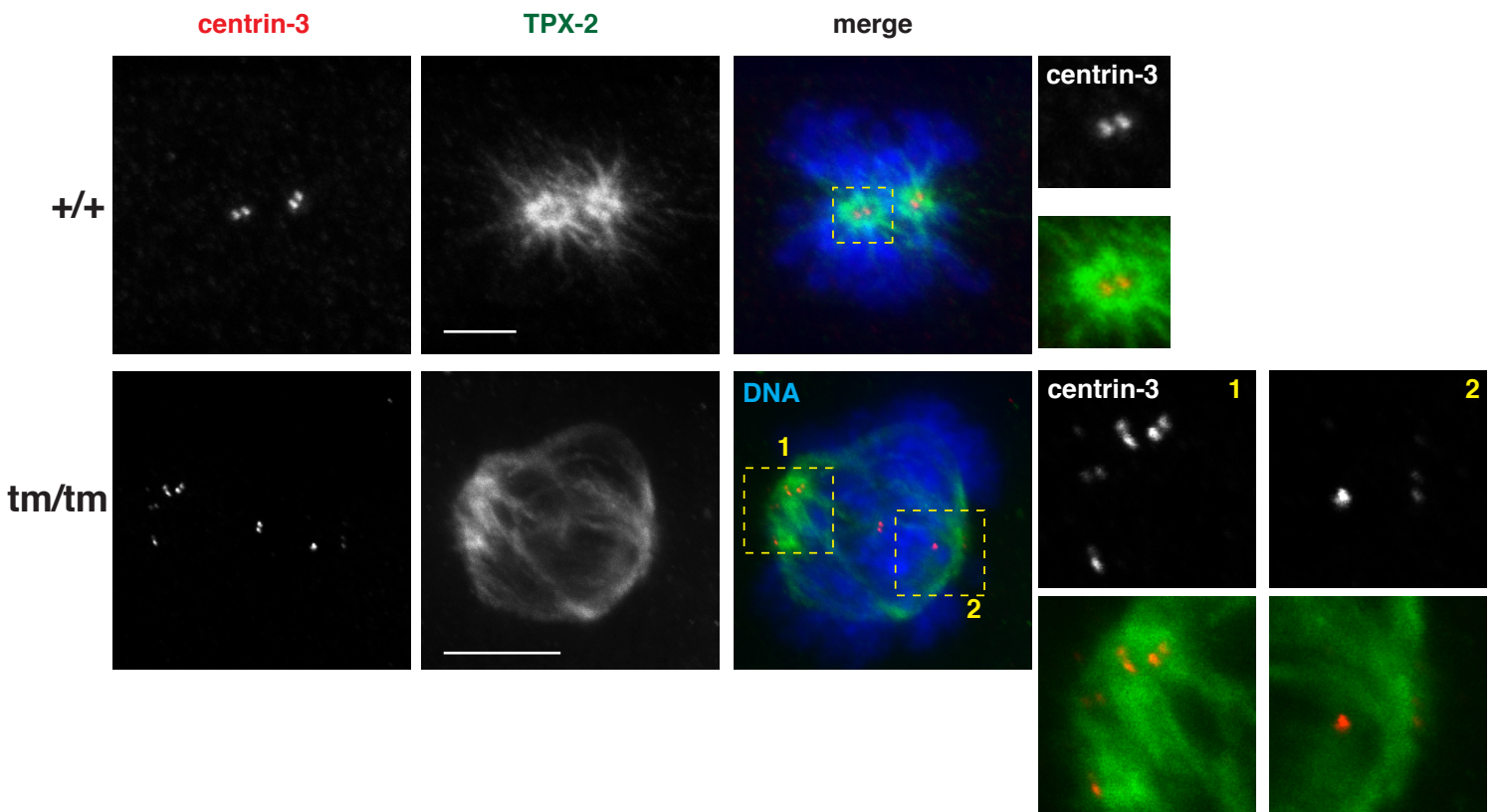

B

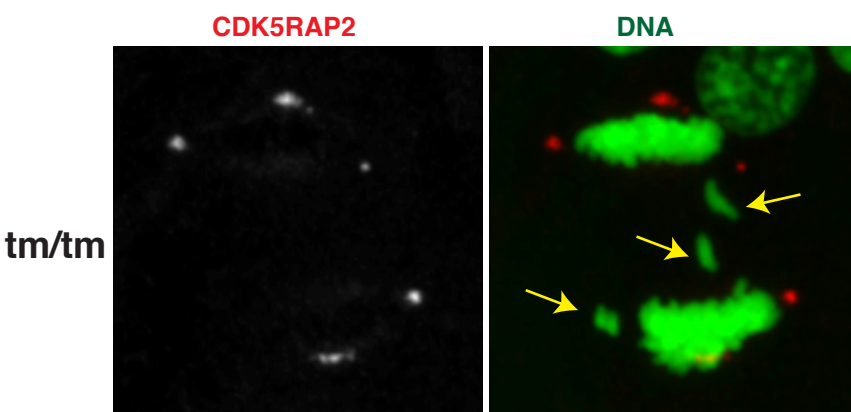

Supplement: Figure S5 — Centrosomal abnormalities of Cenpjtm/tm cells. A. Images show examples of centrin-3 staining in centrosomes of Cenpj+/+ and Cenpjtm/tm mouse embryonic fibroblasts (MEFs). Cells were stained with antibodies against centrin-3 (red in merge) and the mitotic spindle protein TPX-2 (green in merge). DNA is in blue. Framed areas are shown at higher magnification. In Cenpjtm/tm MEFs several centrioles are clustered in a broad spindle pole. In the centre of the spindle two centrioles are visible: these do not associate with a pole and do not seem to nucleate a major microtubule aster, suggesting that these might be part of an inactive centrosome. B. An example for multiple lagging chromosomes in a cell with supernumerary centrosomes. Cells were stained with antibodies against the centrosomal protein CDK5RAP2 (red in merge). DNA is in green. Arrows mark lagging chromosomes. Scale bars are 5 µm. (PDF) [file pgen.1003022.s005.pdf]

A

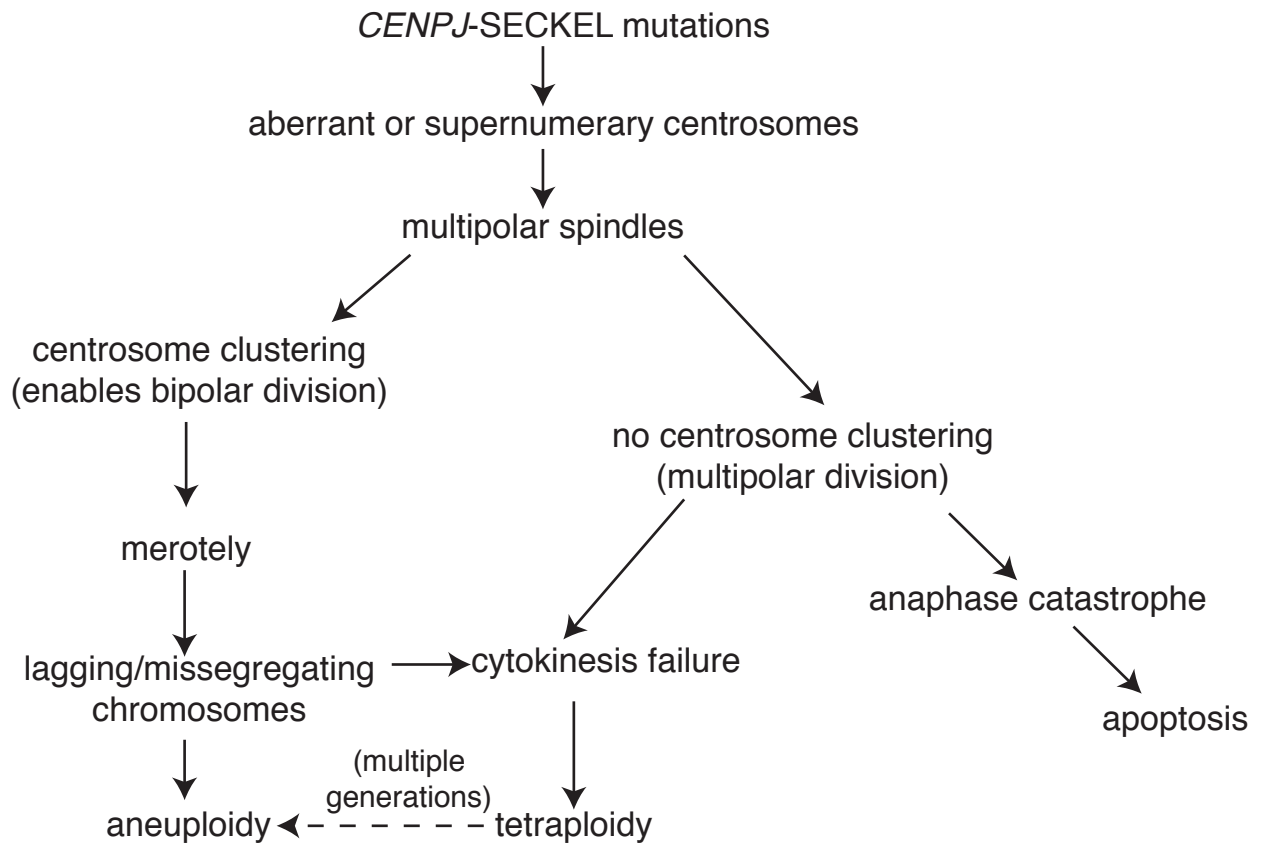

B

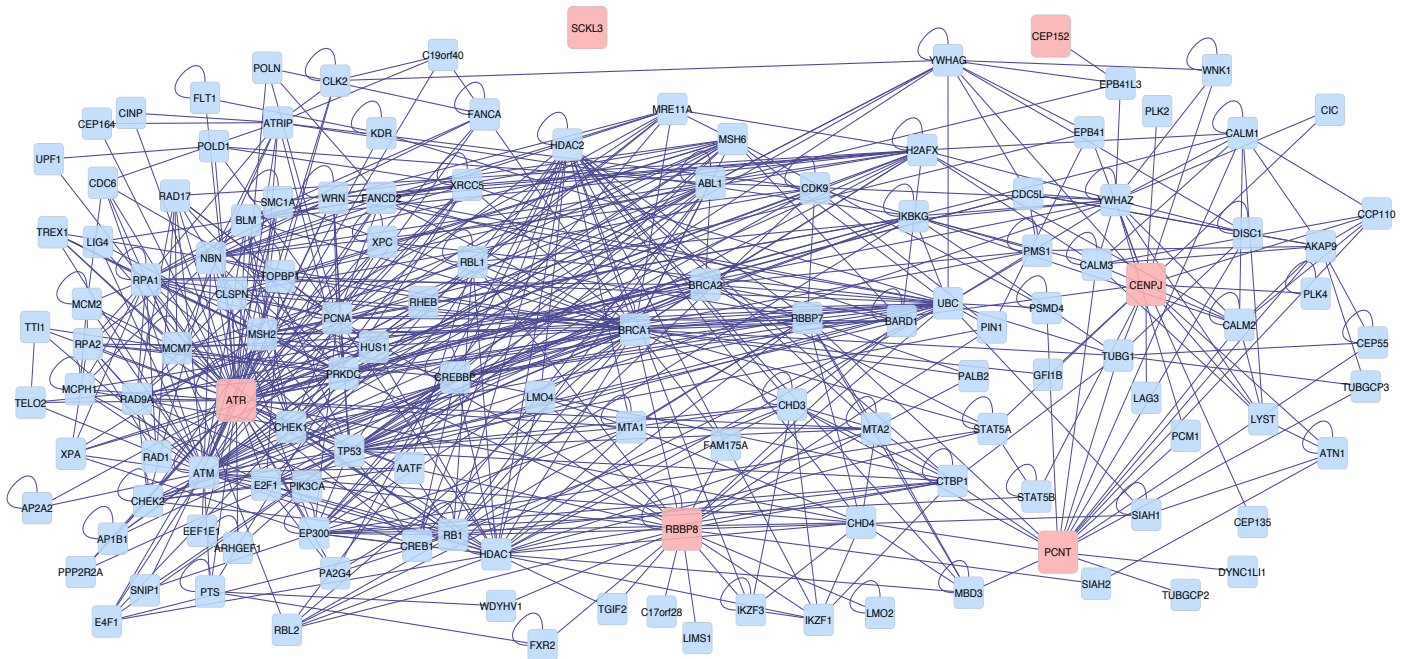

Supplement: Figure S6 — Proposed mechanism of cell death of CENPJ-SECKEL cells and SECKEL protein interaction network. A. Flow diagram to illustrate the sequence of events that may lead to chromosomal instability, polyploidy or cell death of CENPJ-SECKEL cells. Aberrant or supernumerary centrosomes are likely to increase the frequency of multipolar spindle cell intermediates. Clustering centrosomes may enable a bipolar division, however the presence of extra centrosomes increases the frequency of merotelic microtubule-kinetochore attachment errors and leads to lagging chromosomes or missegregation of sister chromatids (aneuploidy). Alternatively, cells with supernumerary centrosomes may undergo a multipolar division; completion of cytokinesis would likely result in non-viable progeny, whereas failure of cytokinesis could result in tetraploidy. It is thought that aneuploid cells may also arise through tetraploid intermediates. B. Using all known Seckel Syndrome associated genes as query, we built a network with 130 genes (nodes) and 665 edges, where the edges represent an experimentally validated protein-protein interaction between the gene products. The pink colored nodes are the known Seckel –syndrome associated genes and the blue colored genes are the ones added by the network expansion analysis. (PDF) [file pgen.1003022.s006.pdf]
