# Supplementary material for: Disruption of Mouse Cenpj, a Regulator of Centriole Biogenesis, Phenocopies Seckel Syndrome
Source: PLoS Genet. 2012 Nov 15;8(11):e1003022. doi: 10.1371/journal.pgen.1003022 (PMC3499256; doi:10.1371/journal.pgen.1003022)
Supplement: Table S2 — Peripheral blood leukocyte analyses: Staining panel 1. A list of antibodies use in the peripheral blood straining 1, the dilution they were used at, and the suppliers of these antibodies. (DOCX) [file pgen.1003022.s008.docx]

**Table S2. Peripheral blood leukocyte analyses: Staining panel 1**

| **Name** | **Dilution** | **Supplier code** |
| --- | --- | --- |
| PerCpCy55-conjugated Rat anti-mouse CD4 (RM4-5) | 1:1000 of 0.2 mg/ml | BD BIOSCIENCE, UK. 550954 |
| PB-conjugated Rat anti mouse CD3 (17A2) | 1:200 of 0.2 mg/ml | eBioscience Ltd, UK. 57-0032-82 |
| APC-conjugated Rat anti-mouse CD25 (PC61) | 1:100 of 0.2 mg/ml | BD BIOSCIENCE, UK. 557192 |
| APC-H7-conjugated Rat anti-mouse CD8a (53-6.7) | 1:200 of 0.2 mg/ml | BD BIOSCIENCE, UK. 560247 |
| PE-conjugated Rat anti-mouse NK1.1 (PK136) (panel 1 and panel 2) | 1:400 of 0.2 mg/ml | BD BIOSCIENCE, UK. 553165 |
| FITC-conjugated Rat anti-mouse CD44 (IM7) | 1:2000 of 0.5 mg/ml | BD BIOSCIENCE, UK. 553133 |
| PE-Cy7-conjugated Rat anti-mouse CD62L (MEL-14) | 1:2000 of 0.1 mg/ml | ab25569 |
