# Supplementary material for: Disruption of Mouse Cenpj, a Regulator of Centriole Biogenesis, Phenocopies Seckel Syndrome
Source: PLoS Genet. 2012 Nov 15;8(11):e1003022. doi: 10.1371/journal.pgen.1003022 (PMC3499256; doi:10.1371/journal.pgen.1003022)
Supplement: Table S3 — Peripheral blood leukocyte analyses: Staining panel 2. A list of antibodies use in the peripheral blood straining 2, the dilution they were used at, and the suppliers of these antibodies. (DOCX) [file pgen.1003022.s009.docx]

**Table S3. Peripheral blood leukocyte analyses: Staining panel 2**

| **Name** | **Stock** | **Supplier code** |
| --- | --- | --- |
| FITC-conjugated Rat anti-mouse IgD  (11-26c.2a) | 1:1000 of 0.5 mg/ml | BD BIOSCIENCE, UK. 553439 |
| PerCpCy55-conjugated Rat anti-mouse Gr1 (RB6-8C5) | 1:800 of 0.2 mg/ml | BD BIOSCIENCE, UK. 552093 |
| PECy7-conjugated Rat anti mouse CD19 (ID3) | 1:1000 of 0.2 mg/ml | BD BIOSCIENCE, UK. 552854 |
| PB-conjugated Rat anti mouse CD11b (M1/70) | 1:800 of 0.1 mg/ml | Caltag, UK. RM2828 |
| Alexa Fluor 700-conjugated Rat anti-mouse CD45 (30F11) (panel 1 and panel 2) | 1:1000 of 0.5 mg/ml | BioLegend, CA, USA. 103128 |
